# Supplementary material for: An expert judgment model to predict early stages of the COVID-19 pandemic in the United States
Source: PLoS Comput Biol. 2022 Sep 23;18(9):e1010485. doi: 10.1371/journal.pcbi.1010485 (PMC9534428; doi:10.1371/journal.pcbi.1010485)
Supplement: S1 Fig — The consent form each expert was presented with and had to agree to before taking part in the survey. This document was shown for every survey. (PDF) [file pcbi.1010485.s001.pdf]

An expert judgment model to predict early stages of the COVID-19 pandemic in the United States

Thomas McAndrew [1*], Nicholas G. Reich [2]

1 College of Health, Lehigh University, Bethlehem, PA, 18015, USA
2 Department of Biostatistics and Epidemiology, University of Massachusetts Amherst School of Public Health and Health Sciences, Amherst, MA, 01003, USA

* mcandrew@lehigh.edu

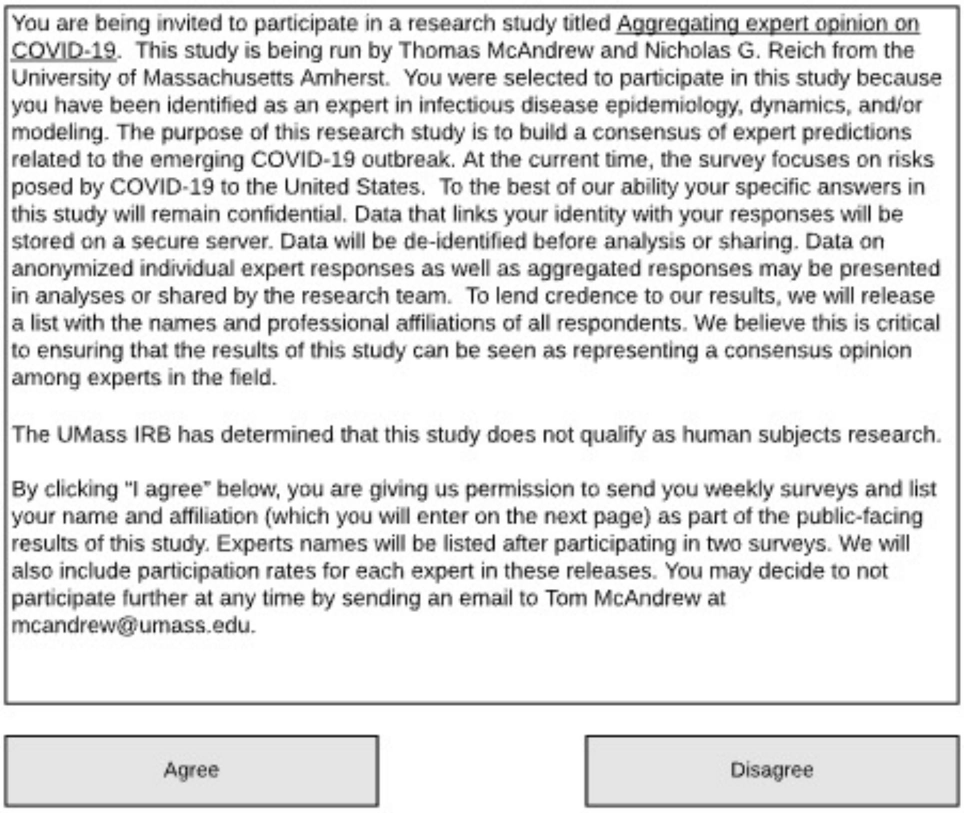

You are being invited to participate in a research study titled Aggregating expert opinion on COVID-19. This study is being run by Thomas McAndrew and Nicholas G. Reich from the University of Massachusetts Amherst. You were selected to participate in this study because you have been identified as an expert in infectious disease epidemiology, dynamics, and/or modeling. The purpose of this research study is to build a consensus of expert predictions related to the emerging COVID-19 outbreak. At the current time, the survey focuses on risks posed by COVID-19 to the United States. To the best of our ability your specific answers in this study will remain confidential. Data that links your identity with your responses will be stored on a secure server. Data will be de-identified before analysis or sharing. Data on anonymized individual expert responses as well as aggregated responses may be presented in analyses or shared by the research team. To lend credence to our results, we will release a list with the names and professional affiliations of all respondents. We believe this is critical to ensuring that the results of this study can be seen as representing a consensus opinion among experts in the field.

The UMass IRB has determined that this study does not qualify as human subjects research.

By clicking "I agree" below, you are giving us permission to send you weekly surveys and list your name and affiliation (which you will enter on the next page) as part of the public-facing results of this study. Experts names will be listed after participating in two surveys. We will also include participation rates for each expert in these releases. You may decide to not participate further at any time by sending an email to Tom McAndrew at mcandrew@umass.edu.

Agree

Disagree

Fig 1. The consent form each expert was presented with and had to agree to before taking part in the survey. This document was shown for every survey.
