# Supplementary material for: An expert judgment model to predict early stages of the COVID-19 pandemic in the United States
Source: PLoS Comput Biol. 2022 Sep 23;18(9):e1010485. doi: 10.1371/journal.pcbi.1010485 (PMC9534428; doi:10.1371/journal.pcbi.1010485)
Supplement: S3 Fig — An example of 10 expert answers to a triplet question, their corresponding triangular probability distributions (TPDs), and an equally-weighted linear pool distribution (black) built from those TPDs. (PDF) [file pcbi.1010485.s003.pdf]

An expert judgment model to predict early stages of the COVID-19 pandemic in the United States

Thomas McAndrew [1*], Nicholas G. Reich [2]

1 College of Health, Lehigh University, Bethlehem, PA, 18015, USA
2 Department of Biostatistics and Epidemiology, University of Massachusetts Amherst School of Public Health and Health Sciences, Amherst, MA, 01003, USA

* mcandrew@lehigh.edu

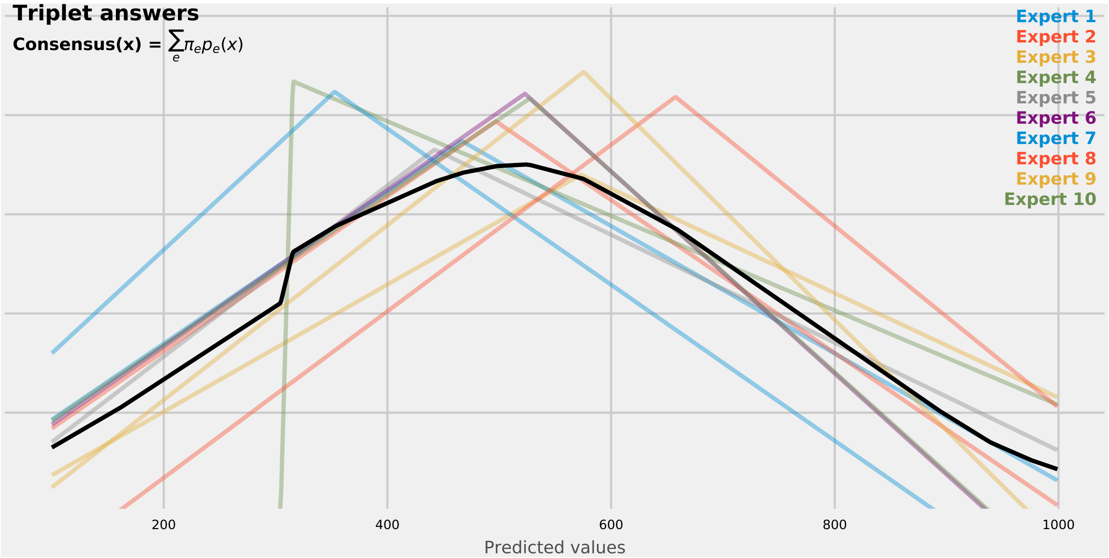

Fig 3. An example of 10 expert answers to a triplet question, their corresponding triangular probability distributions (TPDs), and an equally-weighted linear pool (black) built from those TPDs.
