# Supplementary material for: An expert judgment model to predict early stages of the COVID-19 pandemic in the United States
Source: PLoS Comput Biol. 2022 Sep 23;18(9):e1010485. doi: 10.1371/journal.pcbi.1010485 (PMC9534428; doi:10.1371/journal.pcbi.1010485)
Supplement: S4 Fig — (PDF) [file pcbi.1010485.s004.pdf]

An expert judgment model to predict early stages of the COVID-19 pandemic in the United States

Thomas McAndrew [1*], Nicholas G. Reich [2]

1 College of Health, Lehigh University, Bethlehem, PA, 18015, USA
2 Department of Biostatistics and Epidemiology, University of Massachusetts Amherst School of Public Health and Health Sciences, Amherst, MA, 01003, USA

* mcandrew@lehigh.edu

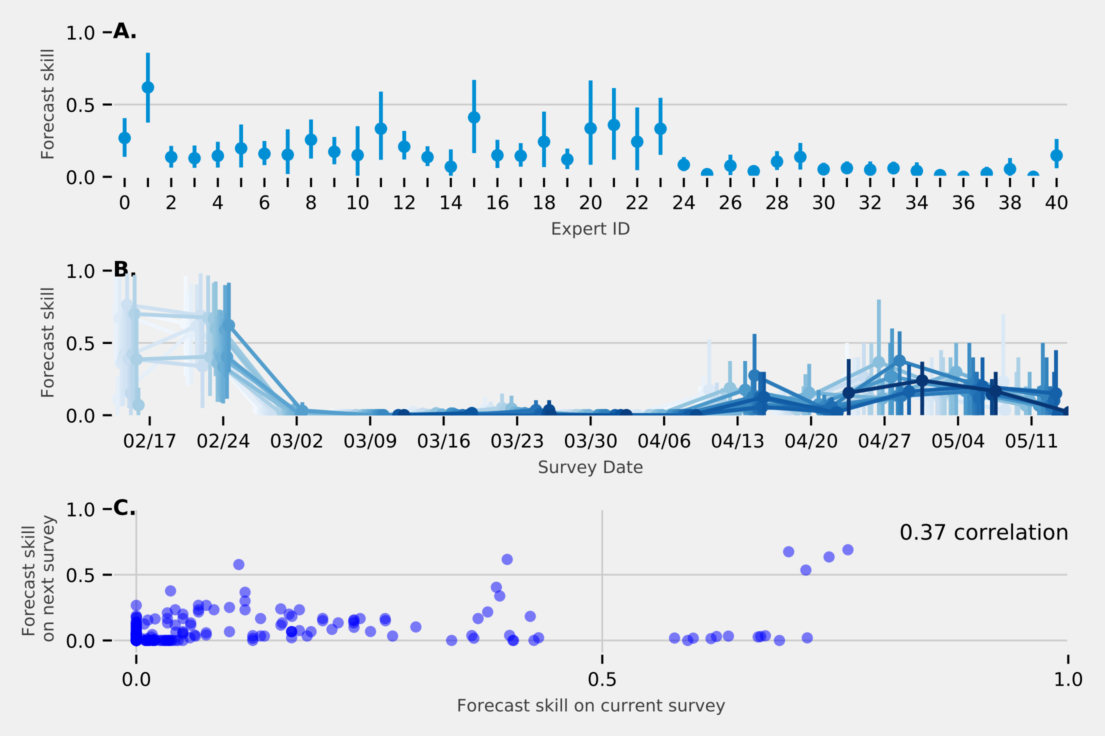

Fig 4. (A.) The average and 95% confidence interval (CI) forecast skill for each expert's answers over the course of the study from mid-February to mid-May. The distribution of forecast skill between experts is on average similar, some experts performing marginally better than others (B.) For each expert, the average and 95% CI forecast skill across surveys. Performance dipped on surveys issued on 3/02 through 4/06. (C.) For every expert, the average forecast skill for the first survey was plotted against the forecast skill of the second survey they completed, the second against third, up until the average forecast skill for the second-to-last and last survey was plotted. Pearson's correlation coefficient was computed. Expert average forecast skill is minimally correlated from one survey to the next and may make it difficult to weight experts based on performance
