# Supplementary material for: An expert judgment model to predict early stages of the COVID-19 pandemic in the United States
Source: PLoS Comput Biol. 2022 Sep 23;18(9):e1010485. doi: 10.1371/journal.pcbi.1010485 (PMC9534428; doi:10.1371/journal.pcbi.1010485)
Supplement: S5 Fig — (PDF) [file pcbi.1010485.s005.pdf]

An expert judgment model to predict early stages of the COVID-19 pandemic in the United States

Thomas McAndrew [1*], Nicholas G. Reich [2]

1 College of Health, Lehigh University, Bethlehem, PA, 18015, USA
2 Department of Biostatistics and Epidemiology, University of Massachusetts Amherst School of Public Health and Health Sciences, Amherst, MA, 01003, USA

* mcandrew@lehigh.edu

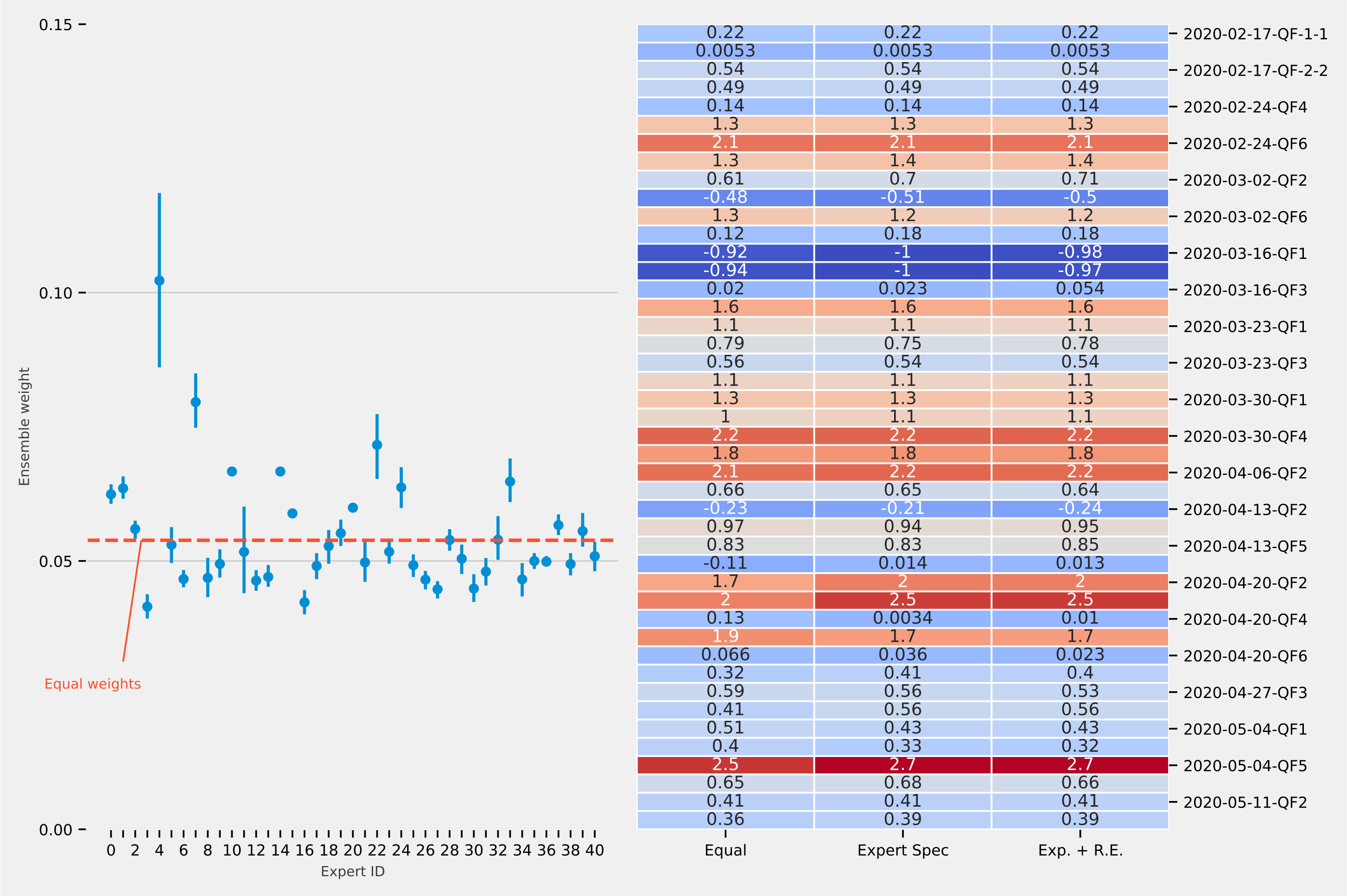

Fig 5. (Left) The average and 95%CI of weights assigned to each expert (from 0-40) by an expert-specific performance weighting over all surveys and questions. The red dotted line denotes the average weight assigned from an equally weighted ensemble. Weights were assigned according to expert's historical performance (the expert specific model). (Right) The difference between log scores and an unskilled forecaster's score for all measurable questions (44 questions) from three linear pools : an equally weighted pool, a linear pool that assigned weights based on expert's past scores (Expert Spec), and a linear pool that assigned weights based on expert's past scores and the relative entropy of expert's answers to the most recent survey questions (Exp + R.E.). Weights assigned to experts based on past performance are not significantly different than the weights assigned by an equally weighted ensemble for the majority of experts. All three linear pools had similar performance to one another.
