# Supplementary material for: An expert judgment model to predict early stages of the COVID-19 pandemic in the United States
Source: PLoS Comput Biol. 2022 Sep 23;18(9):e1010485. doi: 10.1371/journal.pcbi.1010485 (PMC9534428; doi:10.1371/journal.pcbi.1010485)
Supplement: S1 Table — A listing of survey numbers, the date they were issued, information on expert participation, and the database(s) used to collect ground truth. (PDF) [file pcbi.1010485.s006.pdf]

An expert judgment model to predict early stages of the COVID-19 pandemic in the United States

Thomas McAndrew [1*], Nicholas G. Reich [2]

1 College of Health, Lehigh University, Bethlehem, PA, 18015, USA
2 Department of Biostatistics and Epidemiology, University of Massachusetts Amherst School of Public Health and Health Sciences, Amherst, MA, 01003, USA

* mcandrew@lehigh.edu

Survey	Date Issued	Number of participants	Number of questions asked	Sources for truth
1	2020-02-17	15	5	WHO
2	2020-02-24	17	6	WHO
3	2020-03-02	17	6	WHO/CDC
4	2020-03-09	21	6	CDC
5	2020-03-16	19	6	CDC/COVIDTracking
6	2020-03-24	20	6	COVIDTracking
7	2020-03-30	18	7	COVIDTracking
8	2020-04-06	20	5	COVIDTracking
9	2020-04-13	20	6	COVIDTracking
10	2020-04-20	22	6	COVIDTracking/NYC DOH
11	2020-04-27	17	5	COVIDTracking/GA DOH
12	2020-05-04	20	5	COVIDTracking/CDC/TX DOH
13	2020-05-11	17	4	COVIDTracking/JHU CSSE/WA DOH

Table 1. A listing of survey numbers, the date they were issued, information on expert participation, and the database(s) used to collect ground truth. Note that the above includes the number of participants who participated in each survey. Participation is defined as answering one or more questions and several experts did not answer all survey questions.
