# Supplementary material for: An expert judgment model to predict early stages of the COVID-19 pandemic in the United States
Source: PLoS Comput Biol. 2022 Sep 23;18(9):e1010485. doi: 10.1371/journal.pcbi.1010485 (PMC9534428; doi:10.1371/journal.pcbi.1010485)
Supplement: S3 Table — (PDF) [file pcbi.1010485.s008.pdf]

An expert judgment model to predict early stages of the COVID-19 pandemic in the United States

Thomas McAndrew [1*], Nicholas G. Reich [2]

1 College of Health, Lehigh University, Bethlehem, PA, 18015, USA
2 Department of Biostatistics and Epidemiology, University of Massachusetts Amherst School of Public Health and Health Sciences, Amherst, MA, 01003, USA

* mcandrew@lehigh.edu

Caitlin Rivers	Samuel V. Scarpino	Dylan George	Andrew Azman	Jeff Morgan
Marc Lipsitch	Jeffrey Shaman	Roni Rosenfeld	Justin D. Silverman	
Benjamin M. Althouse	Andrew A. Lover	Dr. Caroline Buckee	Helen Jenkins	
John M. Drake	Bryan Lewis	Andreas Handel	Aaron Rumack	
Lauren Gardner	Steven Riley	Mary Bushman	Meagan Fitzpatrick	
Sara Del Valle	Shaun Truelove	William P. Hanage	Stephen Kissler	
Stephen Eubank	Justin Lessler	Srini Venkatramanan	Maimuna Majumder	
Lauren A Castro	Cecile Viboud	Nicholas Reich	Sarah Cobey	
Donald S. Burke, MD	Michael L. Jackson	Natalie Dean	Sebastian Funk	

Table 3. A list of experts who answered at least two surveys (37 out of 41 total experts)
