# Supplementary material for: An expert judgment model to predict early stages of the COVID-19 pandemic in the United States
Source: PLoS Comput Biol. 2022 Sep 23;18(9):e1010485. doi: 10.1371/journal.pcbi.1010485 (PMC9534428; doi:10.1371/journal.pcbi.1010485)
Supplement: S4 Table — (PDF) [file pcbi.1010485.s009.pdf]

An expert judgment model to predict early stages of the COVID-19 pandemic in the United States

Thomas McAndrew [1*], Nicholas G. Reich [2]

1 College of Health, Lehigh University, Bethlehem, PA, 18015, USA
2 Department of Biostatistics and Epidemiology, University of Massachusetts Amherst School of Public Health and Health Sciences, Amherst, MA, 01003, USA

* mcandrew@lehigh.edu

**Dep. Variable:**	Expert specific minus equal weights	**R-squared:**	0.448
**Model:**	OLS	**Adj. R-squared:**	0.429
**Method:**	Least Squares	**F-statistic:**	23.67
**Date:**	Sat, 02 Apr 2022	**Prob (F-statistic):**	3.06e-122
**Time:**	12:20:38	**Log-Likelihood:**	3407.5
**No. Observations:**	1207	**AIC:**	-6733.

**Expert id**	β	**s.e.**(β)	**t**	**P**$>$ |**t**|	[**0.025**	**0.975**]
**0**	0.0086	0.002	3.924	0.000	0.004	0.013
**1**	0.0009	0.006	0.170	0.865	-0.010	0.012
**2**	0.0022	0.002	1.176	0.240	-0.001	0.006
**3**	-0.0126	0.002	-6.551	0.000	-0.016	-0.009
**4**	0.0511	0.002	25.179	0.000	0.047	0.055
**5**	-0.0015	0.003	-0.475	0.635	-0.008	0.005
**6**	-0.0079	0.002	-4.360	0.000	-0.011	-0.004
**7**	0.0244	0.003	7.285	0.000	0.018	0.031
**8**	-0.0063	0.002	-2.602	0.009	-0.011	-0.002
**9**	-0.0060	0.002	-2.745	0.006	-0.010	-0.002
**10**	0	0.008	0	1.000	-0.017	0.017
**11**	-0.0037	0.005	-0.720	0.472	-0.014	0.006
**12**	-0.0085	0.002	-4.628	0.000	-0.012	-0.005
**13**	-0.0080	0.002	-4.406	0.000	-0.012	-0.004
**14**	0	0.008	0	1.000	-0.017	0.017
**15**	-0.0005	0.005	-0.115	0.909	-0.010	0.009
**16**	-0.0101	0.002	-5.038	0.000	-0.014	-0.006
**17**	-0.0065	0.002	-3.065	0.002	-0.011	-0.002
**18**	-0.0030	0.004	-0.757	0.449	-0.011	0.005
**19**	0.0023	0.002	1.254	0.210	-0.001	0.006
**20**	0.0008	0.007	0.107	0.915	-0.014	0.015
**21**	-0.0019	0.004	-0.472	0.637	-0.010	0.006
**22**	0.0124	0.005	2.674	0.008	0.003	0.021
**23**	-0.0050	0.003	-1.496	0.135	-0.012	0.002
**24**	0.0082	0.002	4.044	0.000	0.004	0.012
**25**	-0.0024	0.003	-0.841	0.401	-0.008	0.003
**26**	-0.0071	0.003	-2.652	0.008	-0.012	-0.002
**27**	-0.0085	0.002	-3.794	0.000	-0.013	-0.004
**28**	-0.0008	0.002	-0.346	0.730	-0.005	0.004
**29**	0	0.003	0.001	0.999	-0.006	0.006
**30**	-0.0072	0.003	-2.739	0.006	-0.012	-0.002
**31**	-0.0022	0.003	-0.677	0.499	-0.009	0.004
**32**	0.0031	0.003	0.956	0.340	-0.003	0.009
**33**	0.0112	0.002	5.293	0.000	0.007	0.015
**34**	-0.0033	0.003	-0.970	0.332	-0.010	0.003
**35**	-0.0005	0.005	-0.100	0.921	-0.010	0.009
**36**	-0.0013	0.005	-0.273	0.785	-0.011	0.008
**37**	0.0043	0.005	0.931	0.352	-0.005	0.013
**38**	-0.0012	0.004	-0.317	0.751	-0.009	0.006
**39**	-0.0016	0.006	-0.294	0.769	-0.012	0.009
**40**	-0.0033	0.003	-1.024	0.306	-0.010	0.003

Table 4. Linear regression that compares the weights assigned to each expert using the expert-specific performance weighting and assigning experts equal weights. A positive (negative) estimate of β for expert i indicates an equal weighted ensemble assigned less (more) weight than this performance based ensemble over all surveys.
