# Supplementary material for: An expert judgment model to predict early stages of the COVID-19 pandemic in the United States
Source: PLoS Comput Biol. 2022 Sep 23;18(9):e1010485. doi: 10.1371/journal.pcbi.1010485 (PMC9534428; doi:10.1371/journal.pcbi.1010485)
Supplement: S5 Table — (PDF) [file pcbi.1010485.s010.pdf]

An expert judgment model to predict early stages of the COVID-19 pandemic in the United States

Thomas McAndrew [1*], Nicholas G. Reich [2]

1 College of Health, Lehigh University, Bethlehem, PA, 18015, USA
2 Department of Biostatistics and Epidemiology, University of Massachusetts Amherst School of Public Health and Health Sciences, Amherst, MA, 01003, USA

* mcandrew@lehigh.edu

**Dep. Variable:**	Expert specific + R.E. minus equal weights	**R-squared:**	0.435
**Model:**	OLS	**Adj. R-squared:**	0.416
**Method:**	Least Squares	**F-statistic:**	22.46
**Date:**	Sat, 02 Apr 2022	**Prob (F-statistic):**	1.24e-116
**Time:**	12:20:38	**Log-Likelihood:**	3425.6
**No. Observations:**	1207	**AIC:**	-6769.

**Expert id**	β	**s.e.**(β)	**t**	**P**> |**t**|	[**0.025**	**0.975**]
**0**	0.0061	0.002	2.853	0.004	0.002	0.010
**1**	0.0013	0.005	0.247	0.805	-0.009	0.012
**2**	0.0018	0.002	0.993	0.321	-0.002	0.005
**3**	-0.0126	0.002	-6.668	0.000	-0.016	-0.009
**4**	0.0483	0.002	24.180	0.000	0.044	0.052
**5**	-0.0036	0.003	-1.165	0.244	-0.010	0.002
**6**	-0.0078	0.002	-4.357	0.000	-0.011	-0.004
**7**	0.0237	0.003	7.157	0.000	0.017	0.030
**8**	-0.0072	0.002	-3.007	0.003	-0.012	-0.003
**9**	-0.0043	0.002	-2.003	0.045	-0.009	-8.85e-05
**10**	0	0.008	0	1.000	-0.016	0.016
**11**	-0.0049	0.005	-0.959	0.338	-0.015	0.005
**12**	-0.0078	0.002	-4.308	0.000	-0.011	-0.004
**13**	-0.0074	0.002	-4.136	0.000	-0.011	-0.004
**14**	0	0.008	0	1.000	-0.016	0.016
**15**	-0.0003	0.005	-0.072	0.942	-0.009	0.009
**16**	-0.0105	0.002	-5.290	0.000	-0.014	-0.007
**17**	-0.0057	0.002	-2.727	0.006	-0.010	-0.002
**18**	-0.0031	0.004	-0.810	0.418	-0.011	0.004
**19**	0.0015	0.002	0.808	0.419	-0.002	0.005
**20**	0.0011	0.007	0.153	0.879	-0.013	0.015
**21**	-0.0025	0.004	-0.614	0.539	-0.010	0.005
**22**	0.0124	0.005	2.727	0.006	0.003	0.021
**23**	-0.0051	0.003	-1.553	0.121	-0.012	0.001
**24**	0.0109	0.002	5.459	0.000	0.007	0.015
**25**	-0.0021	0.003	-0.766	0.444	-0.008	0.003
**26**	-0.0066	0.003	-2.507	0.012	-0.012	-0.001
**27**	-0.0076	0.002	-3.459	0.001	-0.012	-0.003
**28**	0.0006	0.002	0.268	0.789	-0.004	0.005
**29**	-0.0009	0.003	-0.272	0.785	-0.007	0.005
**30**	-0.0068	0.003	-2.641	0.008	-0.012	-0.002
**31**	-0.0014	0.003	-0.439	0.661	-0.008	0.005
**32**	0.0025	0.003	0.780	0.435	-0.004	0.009
**33**	0.0116	0.002	5.561	0.000	0.007	0.016
**34**	-0.0049	0.003	-1.493	0.136	-0.011	0.002
**35**	-0.0006	0.005	-0.140	0.888	-0.010	0.008
**36**	-0.0035	0.005	-0.729	0.466	-0.013	0.006
**37**	0.0048	0.005	1.053	0.292	-0.004	0.014
**38**	-0.0008	0.004	-0.224	0.823	-0.008	0.006
**39**	-0.0015	0.005	-0.268	0.789	-0.012	0.009
**40**	-0.0024	0.003	-0.744	0.457	-0.009	0.004

Table 5. Linear regression that compares the weights assigned to each expert using the expert-specific plus relative entropy performance weighting and assigning experts equal weights. A positive (negative) estimate of β for expert i indicates an equal weighted ensemble assigned less (more) weight than this performance based ensemble over all surveys.
