# Supplementary material for: An expert judgment model to predict early stages of the COVID-19 pandemic in the United States
Source: PLoS Comput Biol. 2022 Sep 23;18(9):e1010485. doi: 10.1371/journal.pcbi.1010485 (PMC9534428; doi:10.1371/journal.pcbi.1010485)
Supplement: S6 Table — (PDF) [file pcbi.1010485.s011.pdf]

<table>
<tr><th>Survey issue date</th><th>Question</th><th>Answer type</th></tr>
<tr><td>2020-02-18</td><td>Do you think that the confirmed case count of COVID-19 cases in the US reported by WHO on April 1, 2020 will exceed 100?</td><td>Binary</td></tr>
<tr><td>2020-02-18</td><td>What is the probability that the confirmed case count of COVID-19 cases in the US reported by WHO on April 1, 2020 will exceed 100?</td><td>Binary probabilistic</td></tr>
<tr><td>2020-02-18</td><td>The WHO situation report released on Sunday, 2020-02-16 showed 2 cumulative confirmed cases of COVID-19 in the US with possible or confirmed transmission outside of China. As reported by the WHO this coming Sunday, 2020-02-23, will the number of cumulative confirmed cases in the US with possible or confirmed transmission outside of China exceed 5?</td><td>Binary</td></tr>
<tr><td>2020-02-18</td><td>As reported by the WHO this coming Sunday, 2020-02-23, what is the probability the number of cumulative confirmed cases in the US with possible or confirmed transmission outside of China exceed 5?</td><td>Binary probabilistic</td></tr>
<tr><td>2020-02-18</td><td>What is the smallest, most likely, and largest number of all cumulative confirmed cases (including both imported cases and local transmission) in the US the WHO will report this coming Sunday 2020-02-23?</td><td>Triplet</td></tr>
<tr><td>2020-02-18</td><td>What question(s) do you feel should be asked in next week's survey?</td><td>Text</td></tr>
<tr><td>2020-02-24</td><td>Do you think that the confirmed case count of COVID-19 cases in the US reported by WHO on April 1, 2020 will exceed 100?</td><td>Binary</td></tr>
<tr><td>2020-02-24</td><td>What is the probability that the confirmed case count of COVID-19 cases in the US reported by WHO on April 1, 2020 will exceed 100?</td><td>Binary probabilistic</td></tr>
<tr><td>2020-02-24</td><td>Info: The WHO situation report released on Sunday, 2020-02-23 showed 2 cumulative confirmed cases of COVID-19 in the US with “likely place of exposure” in the US and 14 with the “likely place of exposure” in China.</td><td>Info</td></tr>
<tr><td>2020-02-24</td><td>As reported by the WHO this coming Sunday, 2020-03-01, will the number of cumulative confirmed cases in the US with “likely place of exposure” in the US exceed 5</td><td>Binary</td></tr>
<tr><td>2020-02-24</td><td>As reported by the WHO this coming Sunday, 2020-03-01, what is the probability the number of cumulative confirmed cases in the US with “likely place of exposure” in the US exceed 5?</td><td>Binary probabilistic</td></tr>
<tr><td>2020-02-24</td><td>What is the smallest, most likely, and largest number of all cumulative confirmed cases in the US (with “likely transmission” in China, the US, or elsewhere) that WHO will report this coming Sunday 2020-03-01</td><td>Triplet</td></tr>
<tr><td>2020-02-24</td><td>As of the WHO situation report on Sunday 2020-02-23, 3 countries had reported at least 100 cases of COVID-19. What is the smallest, most likely, and largest number of countries that will have reported at least 100 of cases to WHO (or stopped reporting individual cases due to widespread local transmission) by 2020-03-08</td><td>Triplet</td></tr>
<tr><td>2020-02-24</td><td>What question(s) do you feel should be asked in next week's survey?</td><td>Text</td></tr>
<tr><td>2020-03-02</td><td>Info: The CDC Situation Summary for the COVID-19 outbreak released on Monday, March 2nd, 2020 reported cumulative numbers of 16 confirmed and 27 presumed positive COVID-19 cases in the US. These numbers do not include 48 cases repatriated to the US.</td><td>Info</td></tr>
<tr><td>2020-03-02</td><td>Cumulative confirmed case counts by CDC as of March 2nd, 2020
<table>
<tr><th>Case classification</th><th>Presumptive positive</th><th>Cumulative confirmed cases</th><th>Total</th></tr>
<tr><td>Travel-related</td><td>5</td><td>12</td><td>17</td></tr>
</table></td><td>Info</td></tr>
</table>

Person-to-person spread	22	4	26
Total	27	16	43

2020-03-02	As shown in the table above, the CDC reported 43 total confirmed and presumptive cases of COVID-19 in the US as of Monday, March 2nd. What is the smallest, most likely, and largest number of total confirmed + presumptive cases in the US that CDC will report this coming Monday, March 9th?	Triplet
2020-03-02	What is the smallest, most likely, and largest number of total confirmed + presumptive cases in the US that CDC will report by Monday, March 16th (i.e., 2 weeks from March 2nd)?	Triplet
2020-03-02	Additionally, the CDC reported that 10 states had reported cases as of March 2nd. What is the smallest, most likely, and largest number of states that will have reported cases as of the March 9th update?	Triplet
2020-03-02	As of Monday, March 2nd what percentage of all COVID-19 cases in the US do you believe were reported as confirmed cases? Please indicate the smallest, most likely, and largest percentages below, as integers between 0 and 100.	Triplet
2020-03-02	What percentage of all COVID-19 cases in the US do you believe will be reported as confirmed cases by Monday, March 9th, 2020? Please indicate the smallest, most likely, and largest percentages below, as integers between 0 and 100.	Triplet
2020-03-02	As of the WHO situation report on Sunday, March 1st, seven countries had reported at least 100 laboratory-confirmed cases of COVID-19: China, Korea, Italy, Iran, Japan, Singapore, and France. In the WHO situation report for Sunday, March 8th, what is the smallest, most likely, and largest number of countries that will have at least 100 cases reported, or will have stopped reporting individual cases due to widespread local transmission?	Triplet
2020-03-02	What do you feel is the most important question to ask on next week's survey that could impact decision making in the US?	Text
2020-03-09	Info: The CDC Situation Summary for the COVID-19 outbreak released on Monday, March 9th, 2020 reported cumulative numbers of 423 confirmed and presumed positive COVID-19 cases in the US. These numbers do not include individuals who were repatriated to the US. There is some lag between these case counts and those reported by states. However, we will continue to use these numbers as the official single reference point for case counts in the US.	Info
2020-03-09	As shown in the table above, the CDC reported 423 total confirmed cases of COVID-19 in the US as of Monday, March 9th. What is the smallest, most likely, and largest number of total cases in the US that CDC will report this coming Monday, March 16th?	Triplet
2020-03-09	The CDC reported that 35 states had confirmed cases as of March 9th. What is the smallest, most likely, and largest number of states that will have reported cases as of March 16th?	Triplet
2020-03-09	The CDC defines community spread of COVID-19 as some confirmed cases not knowing how or where they became infected. The CDC currently reports community spread in several regions of the US. Will COVID-19 eventually spread in every state in the US?	Binary
2020-03-09	If community spread of COVID-19 does occur in every state, what is the smallest, most likely, and largest number of weeks until this happens?	Triplet
2020-03-09	As of Monday, March 9th what percentage of all COVID-19 infections in the US (resulting in either symptomatic or asymptomatic illness) do	Triplet

<table>
<tr><td></td><td>you believe were reported as confirmed cases in the CDC tally above? Please indicate the smallest, most likely, and largest percentages below, as values between 0 and 100.</td><td></td></tr>
<tr><td>2020-03-09</td><td>Which month of 2020 will see the highest total number of hospitalizations nationwide in the US for COVID-19 illnesses?</td><td>Triplet</td></tr>
<tr><td>2020-03-09</td><td>Did you find any of the questions above ambiguous or hard to interpret? If so, please explain here. You may also use this space to provide other feedback.</td><td>Text</td></tr>
<tr><td>2020-03-09</td><td>What do you feel is the most important question to ask on next week’s survey that could impact decision making in the US?</td><td>Text</td></tr>
<tr><td>2020-03-16</td><td>Info: The COVID Tracker daily summary released at 4pm on Sunday, March 15th, 2020 reported cumulative numbers of 3512 confirmed (i.e. tested positive) COVID-19 cases in the US.</td><td>Info</td></tr>
<tr><td>2020-03-16</td><td>Data reported by COVID Tracker as of 4pm March 15th, 2020
<table>
<tr><td>Total cases in US</td><td>3512</td></tr>
<tr><td>Total deaths in US</td><td>65</td></tr>
<tr><td>Number of states reporting > 100 cases</td><td>7</td></tr>
</table></td><td>Info</td></tr>
<tr><td>2020-03-16</td><td>As shown in the table above, COVID Tracker reported 3512 total confirmed cases of COVID-19 in the US as of Sunday, March 15th. What is the smallest, most likely, and largest number of total cases in the US that CDC will report this coming Sunday, March 22nd?</td><td>Triplet</td></tr>
<tr><td>2020-03-16</td><td>What is the smallest, most likely, and largest number of total cases in the US that COVID Tracker will report on Sunday, March 29th?</td><td>Triplet</td></tr>
<tr><td>2020-03-16</td><td>As of Sunday, March 15th, COVID Tracker reported 7 states have more than 100 positive cases of COVID-19. These states are: California, Colorado, Florida, Louisiana, Massachusetts, New York, and Washington. What is the smallest, most likely, and largest number of states that will report more than 100 cases this coming Sunday, March 22nd?</td><td>Triplet</td></tr>
<tr><td>2020-03-16</td><td>As of Sunday, March 15th what percentage of all COVID-19 infections in the US (resulting in either symptomatic or asymptomatic illness) are represented by the total case count of 3512 reported by COVID Tracker? Please indicate the smallest, most likely, and largest percentages below, as values between 0 and 100.</td><td>Triplet</td></tr>
<tr><td>2020-03-16</td><td>Over the last 9 seasons, the CDC estimates that the seasonal death toll from influenza outbreaks has ranged from between 11,000 and 95,000. What are the smallest, most likely, and largest number of deaths due to COVID-19 in 2020?</td><td>Triplet</td></tr>
<tr><td>2020-03-16</td><td>What is the probability that there will be a “second wave” of hospitalizations---defined as a distinct peak in national hospitalization rates due to COVID-19---in the fall months (August through December) of 2020? Please take into account the size of the outbreak and preventative measures taken now, or that might be taken in the future. To be a “fall peak” the hospitalization rate need not be higher than a possible “spring peak” it just would need to have a higher value relative to other surrounding months.</td><td>Binary probabilistic</td></tr>
<tr><td>2020-03-16</td><td>Did you find any of the questions above ambiguous or hard to interpret? If so, please explain here. You may also use this space to provide other feedback.</td><td>Text</td></tr>
<tr><td>2020-03-16</td><td>What do you feel is the most important question to ask on next week’s survey that could impact decision making in the US?</td><td>Text</td></tr>
</table>

<table>
<tr><td>2020-03-23</td><td>Info: The COVID Tracker maintains a continually updated database with cumulative numbers of confirmed (i.e. tested positive) COVID-19 cases in the US. They also release fixed daily summaries at 4pm every day.</td><td>Info</td></tr>
<tr><td>2020-03-23</td><td>Data reported by COVID Tracker as of 9am March 23rd, 2020<table><tr><td>Total cases in US</td><td>32,617</td></tr><tr><td>Total deaths in US</td><td>411</td></tr><tr><td>Number of states reporting > 100 cases</td><td>32</td></tr></table></td><td>Info</td></tr>
<tr><td>2020-03-23</td><td>As shown in the table above, COVID Tracker reported 32,617 total confirmed cases of COVID-19 in the US as of Monday, March 23rd at 9am. What is the smallest, most likely, and largest number of total cases in the US that COVID Tracker will report in the daily report this coming Sunday, March 29th?</td><td>Triplet</td></tr>
<tr><td>2020-03-23</td><td>What is the smallest, most likely, and largest number of total cases in the US that COVID Tracker will report on Sunday, April 5th?</td><td>Triplet</td></tr>
<tr><td>2020-03-23</td><td>As of Monday, March 23rd, COVID Tracker reported 32 states and territories (including the 50 states plus the Virgin Islands, Puerto Rico, and DC) have more than 100 positive cases of COVID-19. What is the smallest, most likely, and largest number of states and territories that will report more than 100 cases this coming Sunday, March 29th?</td><td>Triplet</td></tr>
<tr><td>2020-03-23</td><td>As of Monday, March 23rd how many total cumulative SARS-CoV-2 infections (including all symptomatic, subclinical, and asymptomatic infections) have there been in the US?</td><td>Triplet</td></tr>
<tr><td>2020-03-23</td><td>Over the last 9 seasons, the CDC estimates that the seasonal death toll from influenza outbreaks has ranged from between 11,000 and 95,000. What are the smallest, most likely, and largest number of deaths due to COVID-19 in 2020?</td><td>Triplet</td></tr>
<tr><td>2020-03-23</td><td>Which of the next 6 months will see the highest total number of hospitalizations nationwide in the US for COVID-19 illness? Assign a probability to each month representing the likelihood of peak US hospitalizations occurring in that month. Each number must be between 0 and 1 and all numbers provided must sum to 1.</td><td>Categorical probabilistic</td></tr>
<tr><td>2020-03-23</td><td>Did you find any of the questions above ambiguous or hard to interpret? If so, please explain here. You may also use this space to provide other feedback.</td><td>Text</td></tr>
<tr><td>2020-03-23</td><td>What do you feel is the most important question to ask on next week's survey that could impact decision making in the US?</td><td>Text</td></tr>
<tr><td>2020-03-30</td><td>Info: The COVID Tracker maintains a continually updated database with cumulative numbers of confirmed (i.e. tested positive) COVID-19 cases in the US. They also release fixed daily summaries at 4pm every day.</td><td>Info</td></tr>
<tr><td>2020-03-30</td><td>Data reported by COVID Tracker as of 9am March 30th, 2020<table><tr><td>Total cases in US</td><td>141,232</td></tr><tr><td>Total deaths in US</td><td>2,447</td></tr><tr><td>Number of states reporting > 100 cases</td><td>49</td></tr></table></td><td>Info</td></tr>
<tr><td>2020-03-30</td><td>As shown in the table above, COVID Tracker reported 141,232 total confirmed cases of COVID-19 in the US as of Monday, March 30th at 9am. What is the smallest, most likely, and largest number of total</td><td>Triplet</td></tr>
</table>

<table>
<tr><td></td><td>cases in the US that COVID Tracker will report in the daily report this coming Sunday, April 5th?</td><td></td></tr>
<tr><td>2020-03-30</td><td>What is the smallest, most likely, and largest number of total cases in the US that COVID Tracker will report on Sunday, April 12th?</td><td>Triplet</td></tr>
<tr><td>2020-03-30</td><td>As of Monday, March 23rd how many total cumulative SARS-CoV-2 infections (including all symptomatic, subclinical, and asymptomatic infections) have there been in the US?</td><td>Triplet</td></tr>
<tr><td>2020-03-30</td><td>Over the last 9 seasons, the CDC estimates that the seasonal death toll from influenza outbreaks has ranged from between 11,000 and 95,000. What are the smallest, most likely, and largest number of deaths due to COVID-19 in 2020?</td><td>Triplet</td></tr>
<tr><td>2020-03-30</td><td>Which of the next 6 months will see the highest total number of hospitalizations nationwide in the US for COVID-19 illness? Assign a probability to each month representing the likelihood of peak US hospitalizations occuring in that month. Each number must be between 0 and 1 and all numbers provided must sum to 1.</td><td>Categorical probabilistic</td></tr>
<tr><td>2020-03-30</td><td>New confirmed COVID-19 cases in New York State reported by COVID Tracker as of 4pm on each day
<table>
<tr><th>Date</th><th>New cases</th></tr>
<tr><td>3/17/2020</td><td>750</td></tr>
<tr><td>3/18/2020</td><td>682</td></tr>
<tr><td>3/19/2020</td><td>1,770</td></tr>
<tr><td>3/20/2020</td><td>2,950</td></tr>
<tr><td>3/21/2020</td><td>3,254</td></tr>
<tr><td>3/22/2020</td><td>4,812</td></tr>
<tr><td>3/23/2020</td><td>5,707</td></tr>
<tr><td>3/24/2020</td><td>4,790</td></tr>
<tr><td>3/25/2020</td><td>5,146</td></tr>
<tr><td>3/26/2020</td><td>6,447</td></tr>
<tr><td>3/27/2020</td><td>7,377</td></tr>
<tr><td>3/28/2020</td><td>7,683</td></tr>
<tr><td>3/29/2020</td><td>7,195</td></tr>
</table></td><td>Info</td></tr>
<tr><td>2020-03-30</td><td>The table above shows the daily number of new COVID-19 cases in New York State, as reported daily by COVID Tracker for the last two weeks. In New York State, residents are required not to go to work (unless essential personnel) and all non-essential gatherings for any reason are banned. If the current restrictions remain in place, or are made to further restrict social interaction, what are the smallest, most</td><td>Triplet</td></tr>
</table>

<table>
<tr><td></td><td>likely, and largest number of days until a daily number of new confirmed COVID-19 cases in New York State is observed to be below 1,000.</td><td></td></tr>
<tr><td>2020-03-30</td><td>As an expert, we assume that your responses above are a mixture of experience and intuition with knowledge gleaned from specific models that you have developed or seen. What percentage of your response is based on outputs from experience and intuition?</td><td>Binary probabilistic</td></tr>
<tr><td>2020-03-30</td><td>Did you find any of the questions above ambiguous or hard to interpret? If so, please explain here. You may also use this space to provide other feedback.</td><td>Text</td></tr>
<tr><td>2020-03-30</td><td>What do you feel is the most important question to ask on next week’s survey that could impact decision making in the US?</td><td>Text</td></tr>
<tr><td></td><td>Data reported by COVID Tracker as of 9am April 6th, 2020
<table><tr><td>Total cases in US</td><td>333,747</td></tr><tr><td>Total deaths in US</td><td>9,558</td></tr><tr><td>Number of states reporting > 100 cases</td><td>53</td></tr></table></td><td>Info</td></tr>
<tr><td>2020-04-06</td><td>As shown in the table above, COVID Tracker reported 333,747 total confirmed cases of COVID-19 in the US as of Monday, April 6th. What is the smallest, most likely, and largest number of total cases in the US that COVID Tracker will report in the 4pm daily summary on this coming Sunday, April 12th?</td><td>Triplet</td></tr>
<tr><td>2020-04-06</td><td>What is the smallest, most likely, and largest number of total cases in the US that COVID Tracker will report in the 4pm daily summary on Sunday, April 19th?</td><td>Triplet</td></tr>
<tr><td>2020-04-06</td><td>Which of the listed 6 months will see the highest total number of hospitalizations nationwide in the US for COVID-19 illness? Assign a probability to each month representing the likelihood of peak US hospitalizations occurring in that month. Each number must be between 0 and 1 and all numbers provided must sum to 1. (March, April, May, June, July, August)</td><td>Categorical probabilistic</td></tr>
<tr><td></td><td>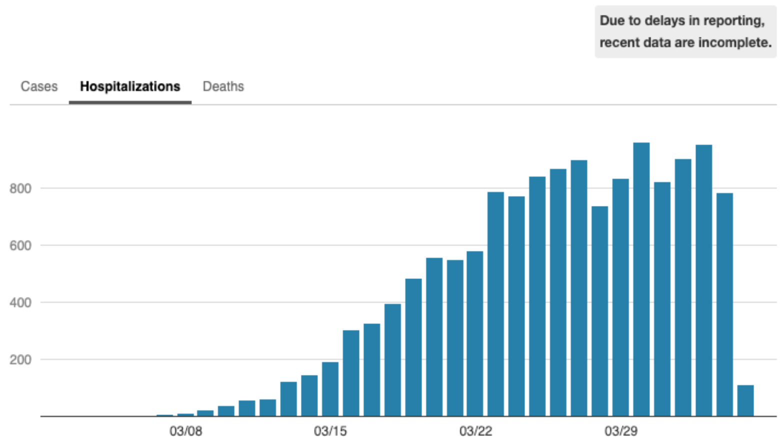
</td><td>Info</td></tr>
<tr><td>2020-04-06</td><td>When will the daily number of new hospital admissions for COVID-19 illness in New York City drop below 200 for the first time since mid-March? Assign a probability to each time period listed below. Each number must be between 0 and 1 and all numbers provided must sum to 1. (The screenshot above is</td><td>Categorical probabilistic</td></tr>
</table>

<table>
<tr><td></td><td>showing latest data from NYC.gov as of 4/6/2020 9am. Note that recent days may be incompletely reported.)

Possible answers:
Before 15 April 2020
Between 15 April 2020 and 30 April 2020
Between 1 May 2020 and 15 May 2020
Between 16 May 2020 and 31 May 2020
Not before 1 June 2020</td><td></td></tr>
<tr><td>2020-04-06</td><td>On the Sunday, April 5 COVID tracker daily report, 332,308 confirmed COVID-19 illnesses were reported in the US. On this same date, how many total cumulative SARS-CoV-2 infections (including all symptomatic, subclinical, and asymptomatic infections) were there in the US?</td><td>Triplet</td></tr>
<tr><td>2020-04-06</td><td>Did you find any of the questions above ambiguous or hard to interpret? If so, please explain here. You may also use this space to provide other feedback.</td><td>Text</td></tr>
<tr><td></td><td>What do you feel is the most important question to ask on next week's survey that could impact decision making in the US?</td><td>Text</td></tr>
<tr><td>2020-04-13</td><td>Data reported by COVID Tracker as of 9am April 13th, 2020
<table><tr><td>Total cases in US</td><td>553,602</td></tr><tr><td>Total deaths in US</td><td>21,984</td></tr><tr><td>Number of states reporting > 1,000 deaths</td><td>3</td></tr></table></td><td>Info</td></tr>
<tr><td>2020-04-13</td><td>
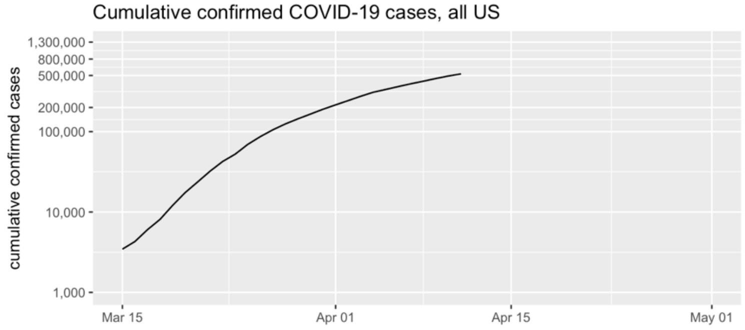

As shown in the table and figure above, COVID Tracker reported 553,602 total confirmed cases of COVID-19 in the US as of Monday, April 13th at 9am. What is the number of total confirmed cases in the US that COVID Tracker will have in the daily report this coming Sunday, April 19th? We have provided a set of ranges of possible confirmed cases. Assign a probability to each bin corresponding to your belief of how many cases will be reported next Sunday. Each number must be between 0 and 1 and all numbers provided must sum to 1.

Less than 700,000 - [0,700,000)
Between 700,000 and 750,000 inclusive - [700,000,750,000]
Greater than 750,000 but less than or equal to 800,000 - (750,000, 800,000]
Greater than 800,000 but less than or equal to 850,000 - (800,000, 850,000]
Greater than 850,000 but less than or equal to 900,000 - (850,000, 900,000]</td><td>Categorical probabilistic</td></tr>
</table>

	Greater than 900,000 but less than or equal to 950,000 - (900,000, 950,000]<br>Greater than 950,000 but less than or equal to 1,000,000 - (950,000, 1,000,000]<br>Greater than 1,000,000 - (1,000,000,)	
2020-04-13	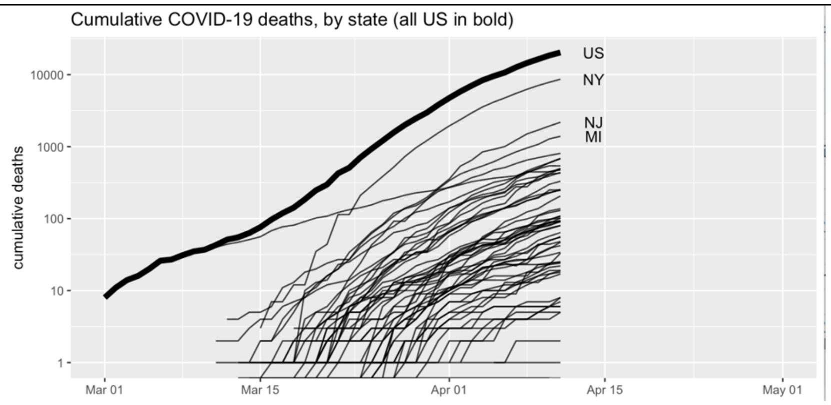<br><br>As shown in the figure above, as of Sunday April 12th, 2020, 3 states had reported over 1,000 COVID-19 deaths (MI, NJ, and NY). How many states will have reported more than 1000 COVID-19 deaths by May 1, according to [COVID Tracker]?<br><br>Greater than 0 and less than 5 - (0,5)<br>Between 5 and 7 inclusive - [5,7]<br>Greater than 7 and less than or equal to 11 - (7,11]<br>Greater than 11 and less than or equal to 13 - (11,13]<br>Greater than 13 - (13,)	Categorical probabilistic
2020-04-13	Which of the next 6 months will see the highest total number of deaths nationwide in the US for COVID-19 illness? Assign a probability to each month representing the likelihood of peak US deaths occurring in that month. Each number must be between 0 and 1 and all numbers provided must sum to 1.<br><br>Prob for April<br>Prob for May<br>Prob for June<br>Prob for July<br>Prob for August<br>Prob for September	Categorical probabilistic
2020-04-13	As of May 1, 2020, what is the smallest, most likely, and largest number of COVID-19 deaths in the US, as reported by COVID Tracker?	Triplet
2020-04-13	As of June 1, 2020, what is the smallest, most likely, and largest number of COVID-19 deaths in the US, as reported by COVID Tracker?	Triplet
2020-04-13	As an expert, we assume that your responses above combine (1) general knowledge about disease dynamics as applied to the ongoing COVID-19 outbreak, including research that you have read or heard about with (2) specific knowledge gleaned from models with which you have been personally involved in developing. What percentage of your response is based on the latter of these: specific models that you have involved in developing?	Continuous

<table>
<tr><td>2020-04-13</td><td>Did you find any of the questions above ambiguous or hard to interpret? If so, please explain here. You may also use this space to provide other feedback.</td><td>Text</td></tr>
<tr><td>2020-04-13</td><td>What do you feel is the most important question to ask on next week’s survey that could impact decision making in the US?</td><td>Text</td></tr>
<tr><td>2020-04-19</td><td>Data reported by COVID Tracker as of 9am April 20th, 2020
<table><tr><td>Total cases in US</td><td>751,062</td></tr><tr><td>Total deaths in US</td><td>35,875</td></tr></table></td><td>Info</td></tr>
<tr><td>2020-04-19</td><td>As shown in the table and figure above, COVID Tracker reported 751,062 total confirmed cases of COVID-19 in the US as of Monday, April 20th at 9am. What is the number of total confirmed cases in the US that COVID Tracker will have in the daily report this coming Sunday, April 26th?

We provided a set of ranges of possible confirmed cases. Assign a probability to each bin corresponding to your belief of how many cases will be reported next Sunday. Each number must be between 0 and 1 and all numbers provided must sum to 1.

Less than 850,000 - [0, 850,000]
Greater than 850,000 and less than or equal to 900,000- (850,000, 900,000]
Greater than 850,000 and less than or equal to 900,000- (900,000, 950,000]
Greater than 950,000 and less than or equal to 1,000,000- (950,000, 1,000,000]
Greater than 1,000,000 and less than or equal to 1,050,000- (1,000,000, 1,050,000]
Greater than 1,050,000 and less than or equal to 1,100,000- (1,050,000, 1,100,000]
Greater than 1,100,000 - (1,100,000,)</td><td>Categorical probabilistic</td></tr>
<tr><td>2020-04-19</td><td>The COVIDtracker reported 35,875 deaths today, April 20th, at 9am. As of Saturday May 9, 2020, how many COVID-19 related deaths will have occurred in the US, as reported by COVID Tracker? Please report a 5th, 50th and 95th percentile, in other words a 90% confidence interval and a median.</td><td>Triplet</td></tr>
<tr><td>2020-04-19</td><td>The COVIDtracker reported 1,296 deaths today, April 20th, at 9am in Louisiana.The COVIDtracker reported 1,290 deaths today, April 20th, at 9am in Illinois. As of Saturday May 9, 2020, how many COVID-19 related deaths will have occurred in Illinois, as reported by COVID Tracker? Please report a 5th, 50th and 95th percentile, in other words a 90% confidence interval and a median.</td><td>Triplet</td></tr>
<tr><td>2020-04-19</td><td>As of Saturday May 9, 2020, how many COVID-19 related deaths will have occurred in Louisiana, as reported by COVID Tracker? Please report a 5th, 50th and 95th percentile, in other words a 90% confidence interval and a median.</td><td>Triplet</td></tr>
<tr><td>2020-04-19</td><td>Over the last 9 influenza seasons, the CDC estimates that the seasonal death toll from influenza outbreaks has ranged from between 11,000 and 95,000. So far 35,875 deaths from COVID-19 have been reported in the US in 2020. How many deaths due to COVID-19 will occur in the US in 2020? Please report a 5th, 50th and 95th percentile, in other words a 90% confidence interval and a median.</td><td>Triplet</td></tr>
<tr><td>2020-04-19</td><td>When will the daily number of new hospital admissions for COVID-19 illness in New York City drop below 200 for the first time since mid-March? Assign a probability to each time period listed below. Each</td><td>Categorical probabilistic</td></tr>
</table>

	number must be between 0 and 1 and all numbers provided must sum to 1. (The screenshot above is showing latest data from NYC.gov as of 4/20/2020 9am. Note that recent days may be incompletely reported.)<br><br>Before 30 April 2020Between 1 May 2020 and 15 May 2020<br>Between 16 May 2020 and 31 May 2020<br>Between 1 June 2020 and 15 June 2020<br>After 15 June 2020	
2020-04-19	A lot of participants have commented that asking a question about intervention effectiveness or the impact of lifting interventions would be valuable. We agree. But we are not sure what to ask or how to ask it in a quantitative way to maximize relevance to ongoing public health operations. We would welcome specific suggestions for how to ask a quantitative question that addresses current or future intervention policies	Text
2020-04-19	Did you find any of the questions above ambiguous or hard to interpret? If so, please explain here. You may also use this space to provide other feedback.	Text
2020-04-27	Info: The COVID Tracker maintains a continually updated database with cumulative numbers of confirmed (i.e. tested positive) COVID-19 cases in the US. They also release fixed daily summaries around 4pm every day.<br>**Data reported by COVID Tracker as of 9:00am April 27th, 2020**<br>**Total cases in the US** 959,056<br>**Total deaths in the US** 49,164	
2020-04-27	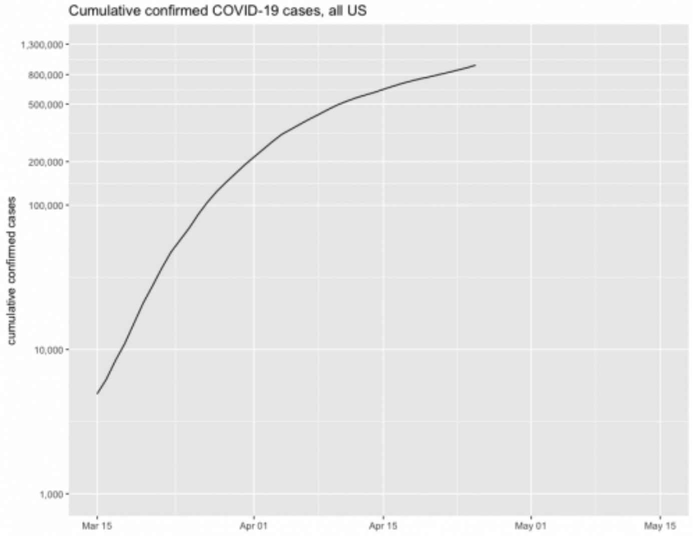<br><br>As shown in the table and figure above, COVID Tracker reported 959,056 total confirmed cases of COVID-19 in the US as of Monday, April 27th at 9am. What is the number of total confirmed cases in the US that COVID Tracker will have in the daily report this coming Sunday, May 3rd (1 week ahead)?<br>We provided a set of ranges of possible confirmed cases. Assign a probability to each bin corresponding to your belief of how many cases will be reported next Sunday. Each number must be between 0 and 1 and all numbers provided must sum to 1.<br><br>Less than or equal to 1,050,000<br>Greater than 1,050,000 and less than or equal to 1,100,000<br>Greater than 1,100,000 and less than or equal to 1,150,000	Categorical probabilistic

	Greater than 1,150,000 and less than or equal to 1,200,000<br>Greater than 1,200,000	
2020-04-27	As of Monday, April 27th how many total cumulative SARS-CoV-2 infections (including all symptomatic, subclinical, and asymptomatic infections) have there been in the US? Please report a 10th, 50th and 90th percentile. In other words give numbers for which you think there is a 1 in 10 chance the truth is below (10th percentile), 1 in 2 chance the truth is below (50th percentile) and 1 in 10 chance the truth is above (90th percentile).<br><br>10th percentile<br>50th percentile (median)<br>90th percentile	Percentiles
2020-04-27	Using data from the COVID tracker daily reports from the past five weeks (starting with the week ending Saturday March 28, through the week ending Saturday April 25), we have seen 1710, 6389, 12027, 13708, and 13788 **new** deaths reported each week in the US.<br><br>Assign probabilities to the following dates, according to when you expect to see the weekly new reported deaths (as calculated using new reported deaths from Sunday through Saturday) first fall below 5,000.<br><br>On Saturday May 2 (from Sunday April 26th) or May 9 (from Sunday May 3rd)<br>On Saturday May 16 or May 23<br>On Saturday May 30 or June 6<br>On Saturday June 6 or June 13<br>After June 20th	Categorical probabilistic
2020-04-27	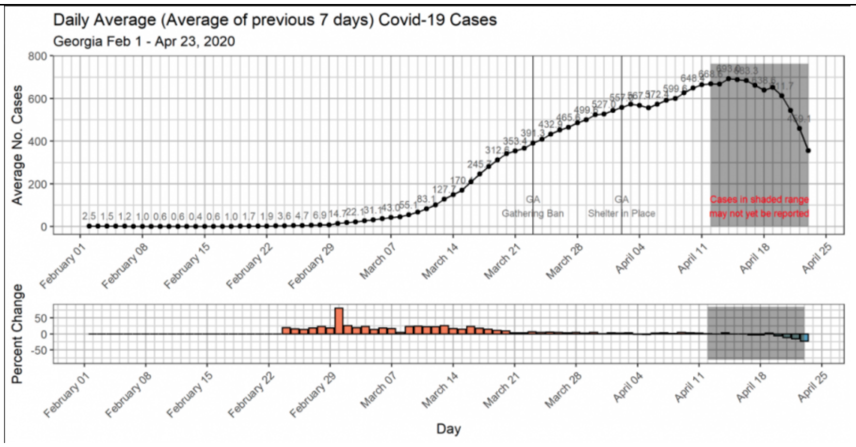<br>*https://dhpexternal.dph.ga.gov/ncovreps/daily_rolling_deaths.png*<br><br>Over the last week, stay-at-home restrictions in Georgia have been lifted and some businesses have reopened. Since April 11, Georgia has reported a rolling 7-day average of just under 700 new confirmed cases per day (see figure above).<br><br>With the knowledge that restrictions may be tightened again or further loosened over the next few weeks, how many new COVID-	Percentiles

	19 cases per day should Georgia expect to see on average during the time-period of May 10-16? Please report a 10th, 50th and 90th percentile.<br><br>10th percentile<br>50th percentile (median)<br>90th percentile	
2020-04-27	Assuming that the restrictions had NOT been loosened and the stay-at-home order had remained in place and businesses remained closed, how many new cases would you have expected Georgia to see per day on average over the time period of May 10-16? Please report a 10th, 50th and 90th percentile.	Triplet
2020-04-27	Did you find any of the questions above ambiguous or hard to interpret? If so, please explain here. You may also use this space to provide other feedback.	Comment
2020-05-04	Info: The COVID Tracker maintains a continually updated database with cumulative numbers of confirmed (i.e. tested positive) COVID-19 cases in the US. They also release fixed daily summaries around 4pm every day.<br>**Data reported by COVID Tracker as of 4:00pm May 3rd, 2020**<br>**Total cases in the US** 1,152,006<br>**Total deaths in the US** 61,868	
2020-05-04	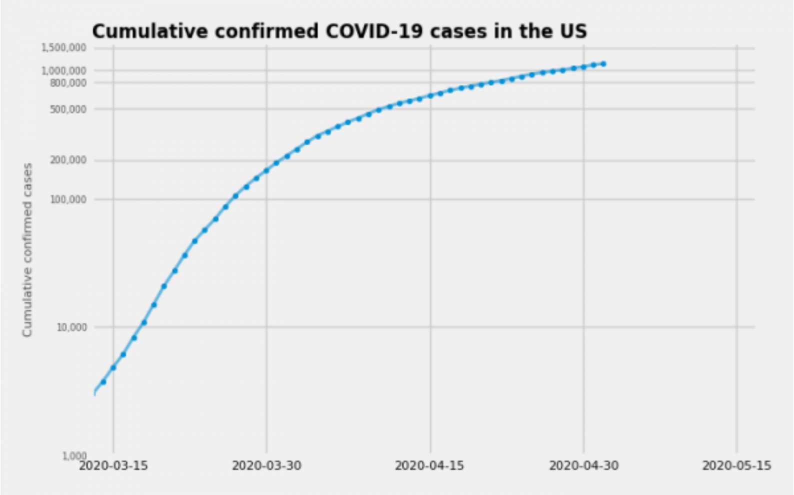<br>*As shown in the table and figure above, COVID Tracker reported 1,152,006 total confirmed cases of COVID-19 in the US as of Monday, May 4th at 9am.*<br><br>*How many total confirmed cases of COVID-19 in the U.S. will The COVID Tracking Project report as of Sunday 10 May 2020?*<br><br>*We provided a set of ranges of possible positive cases. Assign a probability to each bin corresponding to your belief of how many cases will be reported next Sunday. Each number must be between 0 and 1 and all numbers provided must sum to 1.*<br><br>Less than or equal to 1,200,000<br>Between 1,200,000 and 1,250,000, inclusive	Categorical probabilistic

<table>
<tr>
<td></td>
<td>More than 1,250,000 but less than 1,300,000
Between 1,300,000 and 1,350,000, inclusive
More than 1,350,000 but less than 1,400,000
Between 1,400,000 and 1,450,000, inclusive
More than 1,450,000</td>
<td></td>
</tr>
<tr>
<td>2020-05-04</td>
<td>How many US states, territories and jurisdictions (50 states plus District of Columbia, Guam, the Northern Mariana Islands, Puerto Rico, and the U.S Virgin Islands.) will report more new COVID-19 cases for September 2020 than for June 2020?

Fewer than 4
Between 4 and 11
Between 12 and 19
Between 20 and 27
Between 28 and 35
Between 36 and 43
44 or more</td>
<td>Categorical probabilistic</td>
</tr>
<tr>
<td>2020-05-04</td>
<td>

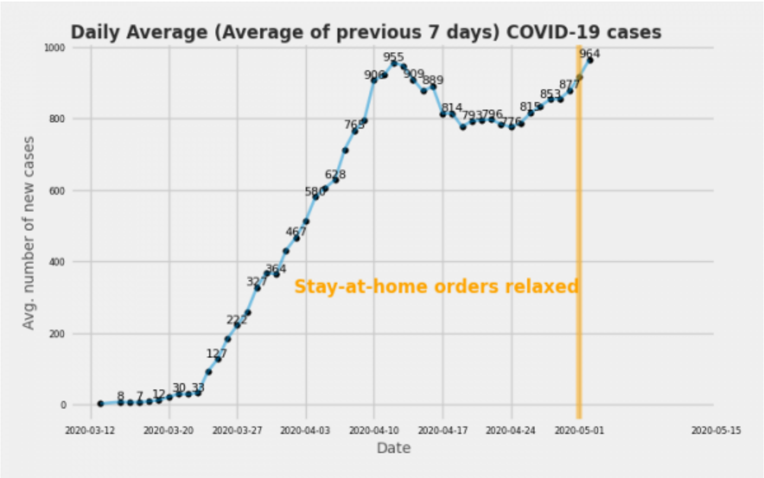

On 1 May 2020, Texas lifted its stay-at-home restrictions and began allowing some businesses to reopen so long as they follow a minimum standard health protocol set by the state and limit their occupancy (link: https://dshs.texas.gov/coronavirus/).

What will the 7 day average number of new positive cases of COVID-19 be in Texas (figure above generated from data reported by the Texas Dept. of Health) for the week ending June 13th 2020, as reported by the Texas Dept. of Health?

Assign a probability to each bin corresponding to your belief of the average number of new positive cases per day in Texas

Less than 675
Between 675 and 900, inclusive
More than 900 but less than 1,350
Between 1,350 and 1,800, inclusive
More than 1,800</td>
<td>Categorical probabilistic</td>
</tr>
<tr>
<td>2020-05-04</td>
<td>Assuming restrictions had NOT been loosened and the stay-at-home order had remained in place and businesses remained closed, what</td>
<td>Categorical probabilistic</td>
</tr>
</table>

	is the 7 day average number of new positive cases of COVID-19 in Texas you would have expected to see for the week ending June 13th 2020?<br><br>Assign a probability to each bin corresponding to your belief of the average number of new positive cases per day in Texas<br><br>Less than 675<br>Between 675 and 900, inclusive<br>More than 900 but less than 1,350<br>Between 1,350 and 1,800, inclusive<br>More than 1,800	
2020-05-04	As of Sunday, May 3, 2020, 61,868 deaths from COVID-19 have been reported in the US in 2020. How many deaths due to COVID-19 will occur in the US in 2020? Please report a 10th, 50th and 90th percentile, in other words a 80% confidence interval and a median.<br><br>10th percentile<br>50th percentile (median)<br>90th percentile	Percentiles
2020-05-11	Info: The COVID Tracker maintains a continually updated database with cumulative numbers of confirmed (i.e. tested positive) COVID-19 cases in the US. They also release fixed daily summaries around 4pm every day.<br>**Data reported by COVID Tracker as of 9:00am May 11th, 2020**<br>**Total cases in the US** 1,322,807<br>**Total deaths in the US** 74,270	Info
2020-05-11	*As shown in the table and figure above, COVID Tracker reported 1,322,807 total positive cases of COVID-19 in the US as of Monday, May 11th at 9am.*<br><br>*What is the number of positive cases in the US that COVID Tracker will have in the daily report this coming Sunday, May 17th?*<br><br>*We provided a set of intervals where the true number of positive cases could fall. Assign a probability to each bin corresponding to your belief of how many cases will be reported next Sunday. Each number must be between 0 and 1 and all numbers provided must sum to 1.*<br><br>*Less than 1,400,000*<br>*Between 1,400,000 and 1,450,000, inclusive*<br>*More than 1,450,000 but less than 1,500,000*<br>*Between 1,500,000 and 1,550,000, inclusive*<br>*More than 1,550,000 but less than 1,600,000*<br>*Between 1,600,000 and 1,650,000, inclusive*<br>*More than 1,650,000*	Categorical probabilistic

2020-05-11	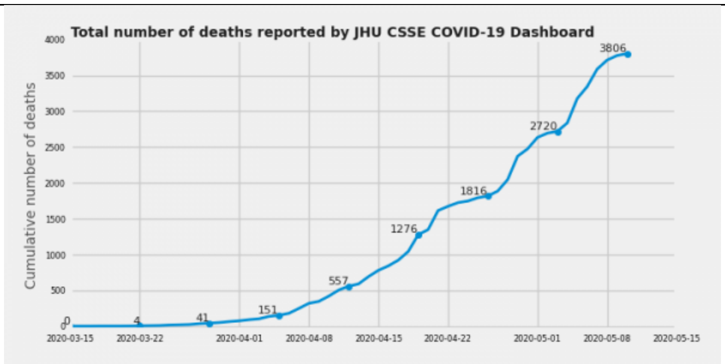<br><br>The state of Pennsylvania ([link to DOH]) has reported 3,806 deaths as of Monday May 11th by the JHU CSSE COVID-19 Dashboard. How many deaths will be reported on Saturday June 13th (4 weeks ahead), as reported by the JHU CSSE Dashboard?<br><br>Please report a 10th, 50th and 90th percentile, in other words a 80% confidence interval and a median.<br><br>10th percentile<br>50th percentile<br>90th percentile	Percentiles
2020-05-11	Stay-home-orders were extended in Washington state until at least May 31st, and the state entered phase one of four phases of their "Safe Start" reopening on May 4. The first phase allows some recreation and construction to reopen and the state will remain in each phase for a minimum of three weeks. Five counties in Washington, with less than 75,000 residents and no new reported cases in the past three weeks, were allowed to begin phase 2. To aid your forecast, the Washington DOH provides a detailed dashboard here.<br><br>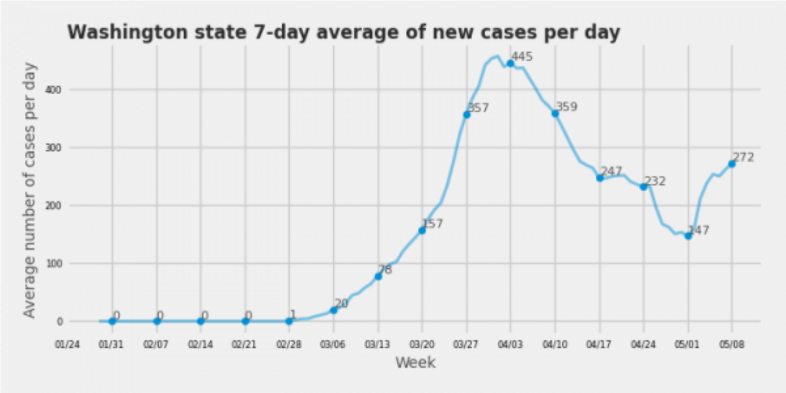<br><br>Given the information above about current orders in Washington state, and accounting for any updates over the coming weeks, what will be the seven day average of new cases per day for the week of June 1, 2020 through June 7, 2020?<br><br>Please report a 10th, 50th and 90th percentile, in other words a 80% confidence interval and a median.<br><br>10th percentile<br>50th percentile<br>90th percentile	Percentiles

2020-05-11	If an accelerated restart in Washington allowed all counties to enter Phase 2 on May 16, 2020 (and was not rescinded before June 1, 2020), what will be the seven day average of new cases per day for the week of June 1, 2020 through June 7, 2020?<br><br>10th percentile<br>50th percentile<br>90th percentile	Percentiles
