# Supplementary material for: An expert judgment model to predict early stages of the COVID-19 pandemic in the United States
Source: PLoS Comput Biol. 2022 Sep 23;18(9):e1010485. doi: 10.1371/journal.pcbi.1010485 (PMC9534428; doi:10.1371/journal.pcbi.1010485)
Supplement: S1 Appendix — (PDF) [file pcbi.1010485.s012.pdf]

An expert judgment model to predict early stages of the COVID-19 pandemic in the United States

Thomas McAndrew [1*], Nicholas G. Reich [2]

1 College of Health, Lehigh University, Bethlehem, PA, 18015, USA
2 Department of Biostatistics and Epidemiology, University of Massachusetts Amherst School of Public Health and Health Sciences, Amherst, MA, 01003, USA

* mcandrew@lehigh.edu

S1 Appendix

A linear pool for a question (q) was created by taking a weighted average of E experts' predictive densities, called a linear pool [1, 2]. We defined a consensus probability distribution for a question q, f_q, as

$$f_q(x) = \sum_{e=1}^{E} \pi(X_e^q, \beta) f_{qe}(x)$$
$$\pi(X_e^q, \beta) = \frac{\exp(X_e^q \beta)}{\sum_e \exp(X_e^q \beta)}$$

where π is the softmax function and where X^q is a design matrix specific to each question asked of experts with one row for each expert and columns for question specific covariates, and X_e^q denotes the eth row of the matrix X^q. The entries in the vector β are estimated parameters associated with each column (covariate) of X^q, and the matrix product $X^q\beta$ determines how weights are assigned to experts. The function $\pi(X_e^q, \beta)$ ensures weights are positive and sum to one, and f_{qe} are predictive densities for question q and expert e.

An equally-weighted linear pool (see Fig. S3. For an example of simulated expert predictions to a triplet question and an equally-weighted consensus) was created by assigning $\pi(X_e^q, \beta) = \dfrac{1}{E_q}$ or one divided by the number of experts who answered questions q.

We also examined two additional models. The first model assigned different weights to each expert based on their past performance.

$$\pi(X_e^q, \beta)_i = x_{i1}\beta_1 + x_{i2}\beta_2 + \cdots x_{iE}\beta_E$$

where β_e is a weight corresponding to expert e, and x_{ie} is equal to 1 when the ith observation corresponds to a prediction made by expert e and is 0 otherwise.

The third model assigned weights to experts based on expert's past performance and on the relative entropy of their probabilistic assignment to a question.

$$\pi(X_e^q, \beta)_i = x_{i1}\beta_1 + x_{i2}\beta_2 + \cdots x_{iE}\beta_E + r_i\beta_r$$

where r_i is the relative entropy of the ith expert's prediction and β_r is the corresponding parameter.

Weights for methods of the above form were estimated using PyMC3 where at any time point we considered all N past true values $t_1, t_2, \cdots, t_N$ i.i.d samples from N

corresponding random variables $t_1 \sim T_1, t_2 \sim T_2, \cdots, t_n \sim T_N$. The following log likelihood was optimized

$$\ell\ell(\beta) = \sum_{i=1}^{N} \log \left[\sum_{e=1}^{E} \pi(X_e^i, \beta) f_{ie}(t_i) \right]$$

Relative entropy (R.E.) is defined as an expert's entropy of the probability distribution assigned to a question divided by the entropy of an unskilled forecaster. Heuristically, higher entropy indicates that a forecasted distribution is less certain and more uniform in its spreading out of probability across possible values. Entropy is defined for a categorical variable as

$$E(x) = -\sum_{c \in C} \log \left[p(x) \right] p(c)$$

where the variable x has C categories and for triplet answers was defined as

$$E(x) = \frac{1}{2} + \log \left[\frac{b-a}{2} \right]$$

where a is the expert's smallest predicted value and b is the largest predicted value. For percentile questions the relative entropy is always one.

Experts who answered a survey for the first time or had no training data were assigned values equal to an unskilled forecaster, for example a relative entropy of one (i.e. the score of an unskilled forecaster). In later surveys, experts were not required to answer all questions, and in these cases, we assigned them the same value as an unskilled forecaster. These assignments enabled all responses to have observed data with which to calculate weights in a given week. Imputed data were only used for training and not used in any statistical comparisons of accuracy.

Training and testing of weighted linear pools All linear pools distributed to public health officials and the public were built using equally weighted linear pools for all responses.

An equally weighted, expert specific weighting, and expert plus relative entropy weighted ensemble model (Table S2) were retrospectively trained on data from the first survey issued on Feb. 17, 2020 up until the last survey issued on May 11. For each week a survey was issued, past data on expert answers and associated log scores (if the truth would have been available at that time) were available for training and used to estimate weights to assign experts who participated in the present survey. Weights were used to form a linear pool and this distribution was scored on the true outcome.

Scores from performance based consensus models were not large enough compared to an equally weighted model to change to a performance based consensus. The minimal difference between an equally weighted and performance based consensus may be due to variability in individual expert performance (Fig. S4). The average and 95CI of weights assigned to each of the 41 experts and a table of log scores for measurable questions for each of the three ensemble models is in Figure S5. A regression analysis comparing the weights assigned by an expert-specific approach and equally weighted approach can be found in table S4 and an analysis that compares the expert specific plus r.e. and equally weights can be found in table S5.

Scores across three consensus models, an equally weighted model, an expert specific model that assigns different weights to each expert, and a model that assigns weights based on individual expert performance and the relative entropy (RE) of their current predictions are similar. Because weights assigned to the expert specific and expert specific plus RE model are similar, relative entropy does not appear to add information about which experts were more predictive than others. Similar scores between the equal

weight and expert specific model could be because an expert's performance on past survey's does not suggest future performance on following surveys (Fig. S4).

Approaches to combine model predictions in the past have had success making in sample and out of sample predictions on a diverse set of datasets [3–7]. The factors that may have prevented our approach from producing improved predictive performance compared to past work could be because of an insufficient number of training examples. Performance could also be impacted by the method used to score predictions or that we scored individual questions rather than assign a score to a experts performance on the entire survey. Additional factors that could impact performance are the specific models that were used to combine predictions, training for experts who made predictions, and varied performance by experts over time.

References

1. Genest C, McConway KJ. Allocating the weights in the linear opinion pool. Journal of Forecasting. 1990;9(1):53–73.

2. Hora SC, Kardeş E. Calibration, sharpness and the weighting of experts in a linear opinion pool. Annals of Operations Research. 2015;229(1):429–450.

3. Polley EC, Van Der Laan MJ. Super learner in prediction. 2010;.

4. Hanea A, Wilkinson DP, McBride M, Lyon A, van Ravenzwaaij D, Singleton Thorn F, et al. Mathematically aggregating experts' predictions of possible futures. PloS one. 2021;16(9):e0256919.

5. Brooks LC, Farrow DC, Hyun S, Tibshirani RJ, Rosenfeld R. Nonmechanistic forecasts of seasonal influenza with iterative one-week-ahead distributions. PLoS computational biology. 2018;14(6):e1006134.

6. Buczak AL, Baugher B, Moniz LJ, Bagley T, Babin SM, Guven E. Ensemble method for dengue prediction. PloS one. 2018;13(1):e0189988.

7. Oidtman RJ, Omodei E, Kraemer MU, Castañeda-Orjuela CA, Cruz-Rivera E, Misnaza-Castrillón S, et al. Trade-offs between individual and ensemble forecasts of an emerging infectious disease. Nature communications. 2021;12(1):1–11.
