# Supplementary material for: An expert judgment model to predict early stages of the COVID-19 pandemic in the United States
Source: PLoS Comput Biol. 2022 Sep 23;18(9):e1010485. doi: 10.1371/journal.pcbi.1010485 (PMC9534428; doi:10.1371/journal.pcbi.1010485)
Supplement: Attachment — Submitted filename: Rerview PCOMPBIOL.pdf [file pcbi.1010485.s013.pdf]

Review PCOMPBIOL-D-21-01928 An expert judgment model to predict early stages of the COVID-19 pandemic in the United States

This article considers COVID-19 expert forecasts and various combinations of forecasts. Forecasts are evaluated against observed outcomes. There are important insights to be drawn from this data and one hopes it can be published. However there are issues with this manuscript: (1) The authors display no awareness of recent literature on expert judgment and its applications including several high visibility applications in the public health area published in PLOS ONE and other good journals. (2) Perhaps as a result there is a discussion of performance weighting in the SI which is impossible to follow, apparently plays no role in the paper proper and appears to give results at odds with the recent literature. These issues are cosmetic and could be easily fixed so that the main paper can be published. The SI is not ready for prime time IMO and should be developed in the light of recent literature and published in a decision theoretic journal. It is not evident to me that this ms belongs in a journal on computational biology, given the wealth of seemingly more appropriate public health journals, but that of course is the editors' call. Given the interest in the COVID data I would favor rapid publication. Details follow.

P.1: The literature cited may be termed archaic. Line 28 and following, the literature on accurately assessing uncertainty is from, 1974,1954, 1985 , cues and target from 1952, 1986, 1996, task complexity 1970,1973. 1976, 1985, cognitive biases from 1974, 1955 and 1997. The unwary reader would conclude that this field has been asleep for 25 years. There is ample recent literature on performance of real experts (not subjects in psychology experiments) which the authors should be able to find.

P.4 lines 100, 117; the format on April 27 experts provided smallest, most likely and largest estimates. This is quite vague and not used in important recent applications. On May 11 it was changed to 10th, 50th and 90th percentiles – more in line with current usage. The reader should be notified why this change was made. Line 129, why is the convex combination of densities called a "consensus distribution"? It suggests experts consented to this which they surely did not. Moreover the "consensus" depends on weights which are not specified. This is misleading labeling. Line 139: the log score is referenced to Gneiting and Raftery who surely did not introduce it, that would be Shuford et al 1965. The important result: that the log rule is the only strictly proper scoring rule which depends only on the probability of the observed outcome (MacCarthy 1956) attributed to Andrew Gleason (of Gleason's theorem). Line 143: Defining "forecast skill" as the exponentiated log of the probability *p(x)* is sure to raise eyebrows of the readers of computational biology. Why not just call it the identity transformation applied to *p(x)? ln(p(x))* is a strictly proper scoring rule but *p(x)* certainly is not. It is called the direct rule and is known to be massively improper: it encourages experts to place unit mass at the mode of their subjective distribution. Moreover these scoring rules were designed for eliciting distributions of individual uncertain quantities. They were not designed for evaluating sets of expert assessments. One simple example will suffice. Consider an expert who assesses the probability of Heads on a coin toss as 0.5. His score for Heads will be the same as his score for tails, namely -0.6913. If he assess 100 tosses his sum score will be -69.13 independent of the frequency of Heads. 100 Heads returns the same score as 50 Heads 50 Tails. There are many such examples. Modern expert judgment theory applies proper scoring rules based on sets of assessments.

There are 11 lines between lines 175 and 176, and one of them should qualify i<V.

Line 178; If these intervals are eg between the 10- 50- and 90-percentiles, then the p_i's won't add to 1.

Line 195, t=0?

Line 334, The study lends strong support for the conclusion "Cultivating and training a pool of expert forecasters should be included as part of larger investments in preparing modeling infrastructure for future outbreaks and pandemics" which I applaud.

Figure 2A, Bedford [43] is presumably not Genest and McConway.

SI (lines not numbered)
Fig 5 is unreadable. The legend doesn't specify which "score" is intended Forecast Skill? relative skill? Rel Abs Err? The legend mentions B, which I can't find in the graph. There is no A. I'm guessing that the numbers (I can only read the ones in black) are the differences between the unskilled skilled forecaster and the various consensus forecasters. If so there is very little daylight between the consensus forecasts, which raises the question why this is presented at all.

Regarding performance based aggregation, if this method had been elaborated in the proper peer reviewed literature to which the reader could refer, then such a summary description might suffice. Absent that, this exposition is inadequate. A regression analysis should AT A MINIMUM include information on R^2, the number of observations and the significance of the covariates. None such is provided. The very small effect of "performance weighting" is probably due to small values of the β's which are not significantly different from zero. In this case the performance weights collapse into equal weights. This supposition is reinforced by the absence of standard information on the regression.

Such regression based approaches have been tried many times and have (as far as I know) not been published because they don't work. Why don't they work? Take a simple example of finding normalized weights w_e that maximize performance of the combination. Experience with real expert data showed that the optimal weights are very unstable, bear little resemblance to an experts' performance and have poor out-of-sample behavior. This model now wants to express the weights as functions of covariates, passing through the softmax function. I would expect this to require much more data than is available here, and would still be subject to the problems mentioned above. Performance weights should themselves be proper scoring rules and should prove their value on diverse real expert data sets (of which there are plenty in the public domain) both in-sample and out-of-sample. Performance weights which meet these requirements have demonstrated clear superiority over equal weighting on many studies – a result at variance with what is reported here.

The importance of enforcing high standards in journals is illustrated by recent gray literature from the CDC where quantile averaging is applied. Quantile averaging of probabilistic forecasts forms a 90% confidence interval whose width is the average of forecasters' 90% confidence

interval widths regardless how far apart these intervals lie. Thus, if one expert has a confidence interval of [0,1] with median ½ while a second has a confidence interval of [9,10] with median 9.5, the quantile average yields a confidence band of [4.5, 5.5] with median 5. Had the authors applied this to diverse real data sets, or consulted the literature where this has already been done, they would see that it leads to poor statistical performance in over half of the cases – much worse than equal weighting. This highlights the risk of launching a new theory based on limited data and also underscores the value of promoting awareness of the relevant literature.

Fig 5. The average and 95%CI of weights assigned to each expert (from 0-40) over all surveys and questions. The red dotted line denotes the average weight assigned from an equally weighted ensemble. Weights were assigned according to expert's historical performance (the expert specific model). (B.) The difference between the consensus score and an unskilled forecaster's score for all measurable questions (40 questions) from three ensemble models: an equally weighted ensemble, an ensemble that assigned weights based on expert's past scores (Expert Spec), and a ensemble that assigned weights based on expert's past scores and the relative entropy of expert's answers to the most recent survey questions (Ex + R.E.). Weights assigned to experts based on past performance are not significantly different than the weights assigned by an equally weighted ensemble. The similarities in weighting translated to similar performance between all three ensembles.
