# Supplementary material for: An expert judgment model to predict early stages of the COVID-19 pandemic in the United States
Source: PLoS Comput Biol. 2022 Sep 23;18(9):e1010485. doi: 10.1371/journal.pcbi.1010485 (PMC9534428; doi:10.1371/journal.pcbi.1010485)
Supplement: Attachment — Submitted filename: Cover_and_reviewer_responses__PCOMPBIOL-D-21-01928.pdf [file pcbi.1010485.s014.pdf]

Thomas McAndrew

Lehigh University
Phone: 1-570-575-4341

Thomas McAndrew · Lehigh University

PLOS: Computational Biology
PCOMPBIOL-D-21-01928
An expert judgment model to predict early stages of the COVID-19 pandemic in the United States

Bethlehem, PA, February 2, 2022

Response to reviewer comments

Dear Dr. Hill, Members of the Editorial Board, and Reviewers,

My coauthor and I thank you for reviewing our work and reviewer comments were well-received.

We addressed all reviewer comments. In particular, we addressed comments from reviewer one about elicitation protocols, changes in how we asked for submissions from experts, and experts analytic versus intuitive thinking. Comments from reviewer two added to the supplemental methods, adding two tables of regression statistics and updates to figures that we appreciate the reviewer commenting on. Comment from reviewer two also contributed to several places in the main text and we included many more citations that this reviewer suggested.

We wish to thank the reviewers for their service and for questions and comments that improved this work. A detailed list of reviewer comments, our response, and the resulting edits to the manuscript are included below. You will find a redline version of the manuscript and supplemental information alongside a revised manuscript and supplement.

We appreciate the opportunity to submit a revised and improved version of the manuscript, and we look forward to hearing from you.

Sincerely,

Thomas McAndrew

Reviewer 1

This is brilliant work and was a real pleasure to read. Thorough efforts evaluating how aggregated expert forecasts perform in real time and how they can be aggregated in ways that facilitates more accurate predictions is crucially important work and no one has done it for COVID-19 more thoroughly than the authors. The extensive longitudinal nature of this work and the authors' willingness to update their protocol as they continued when weaknesses were identified makes it especially valuable. Excellent points made in discussion as well.

We appreciate the reviewers comments and enthusiasm because we too are enthusiastic about this research as well. In particular we thank the reviewer for the positive feedback on our updating the protocol as we changed how we ask questions.

How experts interpreted the phrase "smallest most likely and largest possible number of cases" is hard to know: did they literally interpret that as the smallest/largest possible number of cases such that they would be infinitely flabbergasted if the true number were below/above these extrema? or (probably more likely) did they actually tend to respond with the smallest they thought plausible or even the smallest they thought likely? In the way that the triangular probability densities are constructed it is assumed that the expert meant that there is literally 0 probability of the true value falling below their "smallest" or above their "largest" estimate. This is fine but of course if the experts weren't being quite so literal in their interpretation of "smallest/largest possible" it will result in inadequate allocation of probability mass to outcomes beyond these bounds. It seems possible that the early underestimates might not have seemed quite as extreme if an elicitation protocol were used that took this into account. This should perhaps be briefly discussed. (But I don't want to let the experts off the hook too much for those early predictions as it's clear that they were massive underestimates!)

We agree with the reviewer that we could have made more clear what we meant by the smallest, most likely, and largest. Different experts likely interpreted this question differently and so we moved to a more precise quantile request.

We added a bit of discussion about moving from smallest, most likely, largest to quantiles because experts likely have different views on what these words "smallest", "most likely", and "largest" mean.

" The format for how we asked experts to submit answers changed from smallest, most likely, and largest to a percentile format to more clearly communicate the predictions we meant to elicit from experts. "

Was experts' self-assessed percentage of analytic vs. intuitive thinking predictive of performance? If so mentioning this fact might help inform guidelines for what kinds of thinking experts might consider prioritizing when asked to make probabilistic estimates.

We have included below a plot of the percent of self reported analytic vs intuitive thinking by logarithmic score. Here we assume that an experts self report would apply to the date they answer this question, in the past, and when they answered future surveys. Not everyone answered this question so we lose some experts due to missing data.

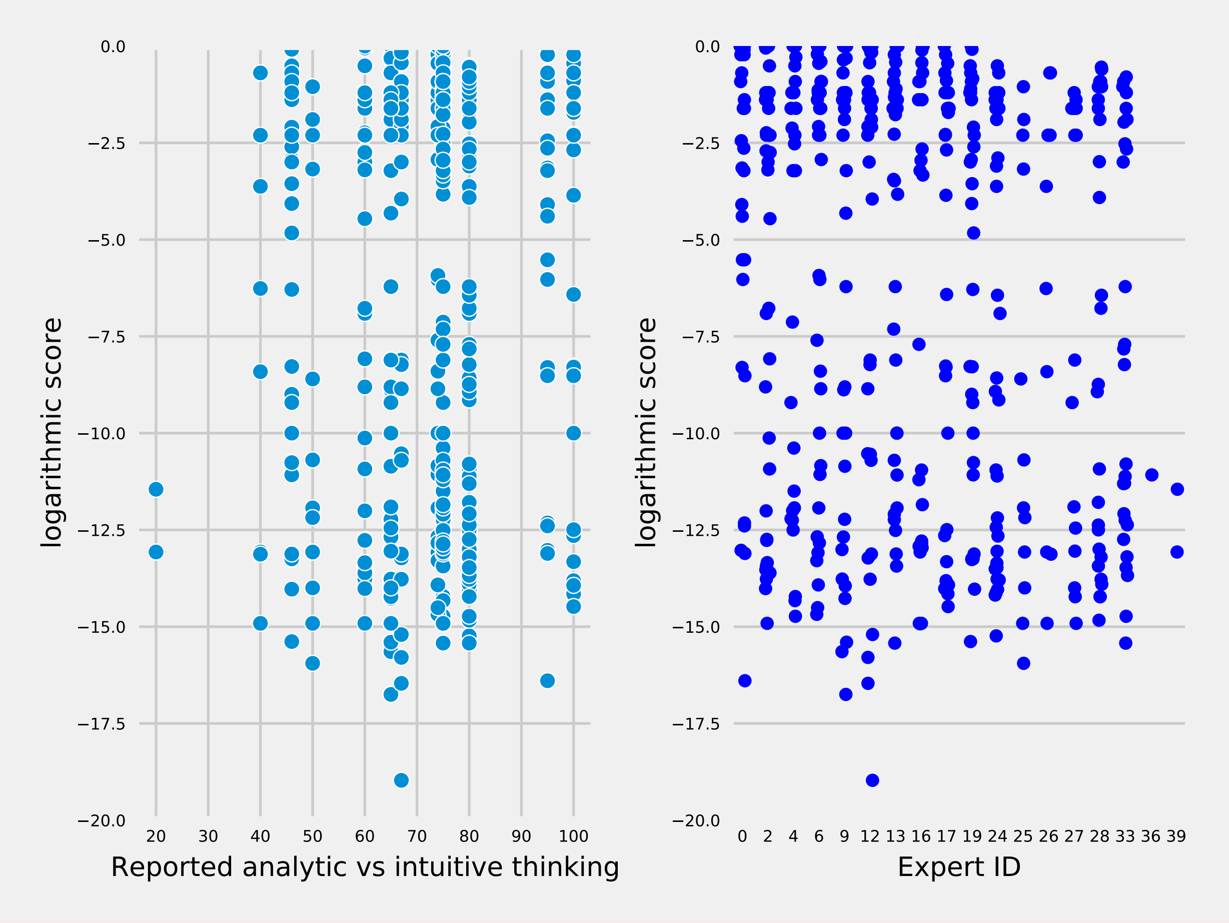

We hoped to see a difference in log score between those who thought more analytic vs intuitive, but from the plot above logarithmic scores varied over the type of thinking. Because the variability in log score for each expert was large, we may not be able to use one variable such as analytic vs intuitive thinking to capture changes in predictive performance.

That said, we would like to explore this in future work and have included a comment in the spirit of what the reviewer suggests in the discussion.

"Insight into the type of thinking experts prioritize when making a prediction and how these modes of cognition lead to more or less accurate predictions may allow us to develop elicitation protocols to either emphasize, or discourage specific approaches to forming a prediction."

It is mentioned that experts spent their careers either working with models of infectious disease dynamics (I'll call these people 'modelers') "and/or the associated policy implications" ('policy wonks'). Was status as modelers vs. policy wonks predictive of performance or is there too little data to say?

This is a good question. We could classify our participants into modelers vs policy experts, however the above plot suggests that the large variability in logscore may make it difficult to predict differences in predictive performance between modelers and policy wonks.

Figure 6 would benefit from an increased resolution if possible.

We agree with the reviewer and increased the resolution.

Despite my critique of asking about the "smallest most likely and largest possible number of cases" above asking participants to consider the extremes of the distribution could slot into an elicitation protocol in other ways. Given that you found that experts gave too-narrow confidence intervals on their early esimates in future studies you might consider a method similar to that described in Ch. 3 of "Developments in Demographic Forecasting" (eds. Mazzuco & Keilman) "Using Expert Elicitation to Build Long-Term Projection Assumptions" (Dion Galbraith and Sirag): (a) Experts are first asked to provide the lower and higher bounds of a range covering nearly all plausible values. Beginning with the contemplation of the extremes of the distribution is an intentional practice used to minimize potential overconfidence (Speirs-Bridge et al. 2010; Sperber et al. 2013; Oakley and O'Hagan 2014; Grigore et al. 2017; Hanea et al. 2018). Indeed asking experts to first provide a single central estimate such as a mean or a median tends to trigger anchoring to that value in subsequent responses. (b) Experts are asked to report how confident they are that the true value will fall within the range they just specified in step 2(a). Allowing experts to determine their own level of confidence has been found to reduce overconfidence in comparison with asking them to identify the low and high bounds of an interval to some predetermined confidence level (Speirs-Bridge et al. 2010). (c) Experts are asked to estimate the median value of the plausible range they provided in step 2(a) so that they expect an equal (50-50) chance that the true value lies above or below the median. (d) The range of values between the lower bound and the median is split in two segments of equal length and the same is done for values between the median and the upper bound. The respondent is then asked to assign to each segment the probability that the true value falls within each of these segments. Note that each half below and above the median has by definition 50% probability of occurrence so it is a matter of redistributing that 50% to each segment. Throughout several "checks" in the form of pop-up warning signs were built-in in order to prevent illogical inputs in various forms."

This is not something that needs to be changed just a thought to consider for future research.

This is an excellent way to elicit a less biased, and likely more adequately confident assessment from each expert. We thank the reviewer for offering the above citations and look forward to reading them to improve how we elicit probabilistic predictive distributions from experts.

In good faith, we included some text in the discussion about elicitation protocols and referred to the above citations.

"There are several elicitation protocols that could have been used to extract less biased and more informative predictions from experts [65,66]."

Reviewer 2

The literature cited may be termed archaic. Line 28 and following the literature on accurately assessing uncertainty is from, 1974, 1954, 1985 , cues and target from 1952, 1986, 1996, task complexity 1970, 1973 , 1976, 1985, cognitive biases from 1974, 1955 and 1997. The unwary reader would conclude that this field has been asleep for 25 years. There is ample recent literature on performance of real experts (not subjects in psychology experiments) which the authors should be able to find.

We appreciate the reviewer bringing to our attention that the references are dated. We agree and have cited recent work (post 2000) alongside these foundational works.

P.4 lines 100 117; the format on April 27 experts provided smallest most likely and largest estimates. This is quite vague and not used in important recent applications. On May 11 it was 10th 50th 90th changed to 10 50 and 90 percentiles – more in line with current usage. The reader should be notified why this change was made.

We have included the following text to help the reader understand why this change was made.

"The format for how we asked experts to submit answers changed from smallest, most likely, and largest to a percentile format to more clearly communicate the predictions we meant to elicit from experts."

Line 129 why is the convex combination of densities called a "consensus distribution"? It suggests experts consented to this which they surely did not. Moreover the "consensus" depends on weights which are not specified. This is misleading labeling.

We thank the reviewer for bringing this to our attention. So as not to mislead we changed the words "consensus distribution" to "linear pool" throughout the manuscript.

We specified the weights for our linear pool in the main text on linear pooling

Linear pooling

A linear pool (f) is a function that takes as input E expert predictions formatted as probability distributions $f_1, f_2, \cdots, f_E$ over a single target value t [49]. We assume the true value t was generated from a random variable T whose distribution we do not know. The linear pool then outputs a probability distribution over t,

$$f(x) = \sum_{e=1}^{E} \pi_e f_e(x) \text{ such that } \sum_{e=1}^{E} \pi_e = 1$$

where π_e is a weight between zero and one assigned to expert e. Because each f_e is a probability distribution the linear pool f is a probability distribution. **For this work we chose to weight all experts equally, assigning a weight of $\pi_e = 1/E$ to each expert** (see S1 Appendix for additional weighting schemes and results).

Line 139: the log score is referenced to Gneiting and Raftery who surely did not introduce it, that would be Shuford et al 1965. The important result: that the log rule is the only strictly proper scoring rule which depends only on the probability of the observed outcome (MacCarthy 1956) attributed to Andrew Gleason (of Gleason's theorem).

We thank the reviewer for pointing us to this literature and have included the citations the reviewer suggests.

Line 143: Defining "forecast skill" as the exponentiated log of the probability p(x) is sure to raise eyebrows of the readers of computational biology. Why not just call it the identity transformation applied to p(x)? ln(p(x)) is a strictly proper scoring rule but p(x) certainly is not. It is called the direct rule and is known to be massively improper: it encourages experts to place unit mass at the mode of their subjective distribution. Moreover these scoring rules were designed for eliciting distributions of individual uncertain quantities. They were not designed for evaluating sets of expert assessments. One simple example will suffice. Consider an expert who assesses the probability of Heads on a coin toss as 0.5. His score for Heads will be the same as his score for tails namely -0.6913. If he assess 100 tosses his sum score will be -69.13 independent of the frequency of Heads. 100 Heads returns the same score as 50 Heads 50 Tails. There are many such examples. Modern expert judgment theory applies proper scoring rules based on sets of assessments.

We appreciate the reviewers comment on the use of the work "forecast skill" and on assessing differences by using the log score. Our usage of terminology and definitions, and the study of scores on an aggregate population are consistent with those in use in the field, including in work from PLOS Comp Bio and Nature.

Reich NG, McGowan CJ, Yamana TK, Tushar A, Ray EL, et al. (2019) Accuracy of real-time multi-model ensemble forecasts for seasonal influenza in the U.S.. PLOS Computational Biology 15(11): e1007486. https://doi.org/10.1371/journal.pcbi.1007486

McGowan, C.J., Biggerstaff, M., Johansson, M. et al. Collaborative efforts to forecast seasonal influenza in the United States, 2015–2016. Sci Rep 9, 683 (2019). https://doi.org/10.1038/s41598-018-36361-9

There are 11 lines between lines 175 and 176 and one of them should qualify i<V

We thank the reviewer for this comment and included the above qualification that $i < V$

Line 178; If these intervals are eg between the 10- 50- and 90-percentiles then the pi's won't add to 1.

We clarified, revised the text so that it is clearer for the reader how they can use linear interpolation and root finding to estimate quantiles.

Given a set of N bins $[x_0, x_1], [x_0, x_1], \cdots, [x_{N-1}, x_N]$ with corresponding probabilities $p_1, p_2, \cdots, p_N$ over a target that takes nonnegative values, quantiles can be imputed by first generating a function f that is a linear interpolant of (x,y) pairs $(x_0, 0), (x_1, P_1), (x_2, P_2), (x_3, P_3), \cdots, (x_N, 1)$, where i ranges from 0 to N and P_i is the cumulative sum of all probabilities with index i or smaller $P_i = p_1 + p_2 + \cdots + p_i$. To compute a quantile q we can find the root of the function $F(x) = f(x) - q$ using the Newton-Raphson algorithm (or any standard root finding procedure).

Line 195 t=0?

We clarified in the text the domain of possible values that the relative absolute error can take.

The relative absolute error is a function that takes as input a point prediction (p) and ground truth value (t) and outputs the absolute difference between the point prediction and ground truth value divided by the ground truth value

$$\text{Rel.Abs.Err}(p, t) = \left| \frac{p}{t} - 1 \right|$$

where the vertical bars indicate the absolute value. The domain of this function is defined only for positive values of t (i.e. $t > 0$).

Line 334 The study lends strong support for the conclusion "Cultivating and training a pool of expert forecasters should be included as part of larger investments in preparing modeling infrastructure for future outbreaks and pandemics" which I applaud.

We too agree that this is an important step forward for outbreak and pandemic preparedness.

Figure 2A Bedford 43 is presumably not Genest and McConway.

We thank the reviewer for pointing this out and corrected the citation.

Fig 5 is unreadable. The legend doesn't specify which "score" is intended Forecast Skill? relative skill? Rel Abs Err? The legend mentions B which I can't find in the graph. There is no A. I'm guessing that the numbers (I can only read the ones in black) are the differences between the unskilled skilled forecaster and the various consensus forecasters. If so there is very little daylight between the consensus forecasts which raises the question why this is presented at all.

We improved the visibility of Fig 5 in the supplement, corrected the figure legend, and clarified what scores we are evaluating. The goal of this analysis was to show the similarities in scores between the three linear pooling methods.

Regarding performance based aggregation if this method had been elaborated in the proper peer reviewed literature to which the reader could refer then such a summary description might suffice. Absent that this exposition is inadequate. A regression analysis should AT A MINIMUM include information on R the number of observations and the significance of the covariates. None such is provided. The very small effect of "performance weighting" is probably due to small values of the β's which are not significantly different from zero. In this case the performance weights collapse into equal weights. This supposition is reinforced by the absence of standard information on the regression.

We thank the reviewer for asking for additional citations and a table of estimated parameters from the three models we chose to implement. We have included citations that reference linear pooling methodology.

To address the second reviewers point, we included two tables in the supplement that report the estimated β parameters, standard errors, t-statistic, pvalue, 95% confidence intervals, R^2 and adjusted R^2 values. The first model that was fit is

$$y_i \sim \mathcal{N}\left(\beta_0 + \sum_{e=1}^{40} \beta_e E_{i,e}, \sigma^2\right) \tag{1}$$

where y_i is the observed difference in expert-specific weighting and equal weights, and $E_{i,e}$ takes the value one when y_i refers to expert e and 0 otherwise. Our second model was the same except one difference: y_i is the observed difference in expert-specific plus R.E. weighting minus equal weights.

The reviewer is correct that the performance-weighted beta parameters do not differ much from what would be assigned had we picked equal weights.

Such regression based approaches have been tried many times and have (as far as I know) not been published because they don't work. Why don't they work? Take a simple example of finding normalized weights we that maximize performance of the combination. Experience with real expert data showed that the optimal weights are very unstable bear little resemblance to an experts' performance and have poor out-of-sample behavior. This model now wants to express the weights as functions of covariates passing through the softmax function. I would expect this to require much more data than is available here and would still be subject to the problems mentioned above. Performance weights should themselves be proper scoring rules and should prove their value on diverse real expert data sets (of which there are plenty in the public domain) both in-sample and out-of-sample. Performance weights which meet these requirements have demonstrated clear superiority over equal weighting on many studies – a result at variance with what is reported here.

We thank the reviewer for this comment. We feel that excluding our work on a regression based weighting approach because it did not improve out of sample performance would support negative publication bias. To soften the results that this approach did not work and to place it in context of other approaches, we included a citation that points to diverse real expert datasets where a regression based approach has proven useful and to a recent methods paper for approaches to combine human judgement.

"Regression approaches in the past have had success making in sample and out of sample predictions on a diverse set of datasets [3,4]. The factors that may have prevented our approach from producing improved predictive performance could be because of not enough training examples, training for experts who made predictions, and varied performance by experts over time. "

The importance of enforcing high standards in journals is illustrated by recent gray literature from the CDC where quantile averaging is applied. Quantile averaging of probabilistic forecasts forms a 90% confidence interval whose width is the average of forecasters' 90% confidence interval widths regardless how far apart these intervals lie. Thus if one expert has a confidence interval of [0, 1] with median 1/2 while a second has a confidence interval of [9,10] with median 9.5 the quantile average yields a confidence band of [4.5 , 5.5] with median 5. Had the authors applied this to diverse real data sets or consulted the literature where this has already been done they would see that it leads to poor statistical performance in over half of the cases – much worse than equal weighting. This highlights the risk of launching a new theory based on limited data and also underscores the value of promoting awareness of the relevant literature.

We appreciate the reviewers criticism of quantile averaging. We agree that quantile averaging can at times result in non-sensical intervals. That said, we offer the following works, one authored by Winkler and the second Busetti, which compared quantile averaging to probability averaging. Both works show that quantile averaging provides comparable, if not at times improved, results. No doubt, this is an interesting and long lasting debate which the present manuscript does not intend to address.

Kenneth C. Lichtendahl, Jr., Yael Grushka-Cockayne, Robert L. Winkler, (2013) Is It Better to Average Probabilities or Quantiles?. Management Science 59(7):1594-1611. https://doi.org/10.1287/mnsc.1120.1667

Busetti, F., 2017. Quantile aggregation of density forecasts. Oxford Bulletin of Economics and Statistics 79, 495–512
