# Supplementary material for: An expert judgment model to predict early stages of the COVID-19 pandemic in the United States
Source: PLoS Comput Biol. 2022 Sep 23;18(9):e1010485. doi: 10.1371/journal.pcbi.1010485 (PMC9534428; doi:10.1371/journal.pcbi.1010485)
Supplement: Attachment — Submitted filename: Response2Review_expertpaper_round2__PCOMPBIOL-D-21-01928.pdf [file pcbi.1010485.s015.pdf]

Reviewer 1

Thank you! I'm satisfied with the way all of my comments were addressed. Congrats again on a nice piece of work.

We thank the reviewer again for constructive feeback that improved this work.

Reviewer 2

Most of the cosmetic issues in the main text have been satisfactorily addressed. The SI is still unacceptable. The more I try to understand the more confusing it becomes. "Consensus distribution" has been replaced by "linear pool" in the main text but not in the SI. There are 40 experts in tables S4 and S5 there are 41 experts on SI p. 7 and in the caption to Fig 5 there are 36 on github. Fig 5 caption says there are 40 "measurable questions" in the right graph where I count 44.

We thank the reviewer for adding additional comments that we can address in the SI.

(i) We replaced the phrase "Consensus distribution" with "linear pool" in the SI.

(ii) The number of experts in the github, main text, and SI are consistent. In the Github we included 37 individual experts who answered two or more surveys. Those that answered one survey were not included by name in the Github. Please see the main text section on Recruitment of Experts. To clarify, we added to this section on recruitment:

"Experts could participate in our surveys after reading and agreeing to a consent document S1 Fig. The consent states that after an expert completed two surveys their name and affiliation would be included in public-facing summaries. The consent form also said that public releases of the data would ensure individual expert responses would remain unidentifiable. A list of experts **who participated in two or more surveys** can be found in the data repository [13] and in Table S3 in the supplement."

We removed the intercept from S4 and S5 so that both tables now include 41 (instead of 40) estimates of weights.

(iii) The caption for Fig 5. was corrected.

p.7 SI says "Experts who answered a survey for the first time or had no training data were assigned values equal to an unskilled forecaster for example a relative entropy of one (i.e. the score of an unskilled forecaster). In later surveys experts were not required to answer all questions and in these cases we assigned them the same value as an unskilled forecaster. These assignments enabled all responses to have observed data with which to calculate weights in a given week." So some of the experts' assessments are just invented? How many?

To allow us to include an expert in training of a performance based ensemble who did not answer previous surveys we performed statistical imputation. The goal of statistical imputation is not to "invent data". Instead the goal is to replace missing data with reasonable estimates so that non-missing data can be included in analysis.

Expert assessments were imputed to train the model and not imputed when comparisons of accuracy are made. To make this point clearer we added to the SI on page 7:

"Imputed data were only used for training and not used in any statistical comparisons of accuracy."

SI tables 4 and 5 give 1207 as "Nr Observations"; presumably that means 1207 assessments of some question by some expert. Table 1 lists the number of experts answering each question. Summing the products (experts * questions) gives 1370.

Table 1 lists the number of experts who participated in each survey, not the number of experts who answered all questions asked. We clarify this point in the table caption.

"Note that the above includes the number of participants who participated in each survey. Participation is defined as answering one or more questions and several experts did not answer all survey questions."

The tables S4 and S5 are hard to interpret without some explanation of what the intercept and the beta's represent. I don't understand why only 4 of the expert betas are positive in each table. Half of the CI's in S5 include zero (in S4 13) meaning that the betas for "expert specific minus equal weights" are not significantly different from zero? Interpretation?

We agree with the reviewer that including an intercept made the table difficult to interpret. Instead, we have removed the intercept from tables S4 and S5, and included additional text to describe the meaning of the β estimates in the caption:

"A positive (negative) estimate of β for expert i indicates an equal weighted ensemble assigned less (more) weight than this performance based ensemble over all surveys." .

SI p.7: "Regression approaches in the past have had success making in sample and out of sample predictions on a diverse set of datasets (3 4). Neither of these references have anything to do with regression. Perhaps the authors confuse the linear pool with linear regression? In any case the differences here between performance weights and equal weights are extremely small. Since this is strongly at variance with a wealth of literature (google "performance weights expert judgment") including recent pubs in PNAS PLOS ONE (WHO) and Emerging Infection Diseases (CDC) the reader deserves some explanation. The factors mentioned on p 7 haven't plagued other approaches. Perhaps the problem lies with the scoring variable. Indeed rewarding honesty is not the same as rewarding goodness as my little counter example pointed out. You don't address counter examples by citing other people making the same mistakes.

We added to the SI 5 references that better relate to combination approaches in forecasting and prediction.

We agree that the difference between performance based and equally weighted ensembles are small. This is why we included this detailed analysis in the supplementary and highlight that there exist small differences in the main manuscript.

The example of 100 coin tosses does not relate well to this work. We did not ask experts a long list of binary questions. The majority of questions we asked experts had many more options than two. The spirit of that comment may be about the difference between many assessments of an expert on individual questions versus a single assessment over an expert's performance on all questions asked in the survey. To address the above comments we added to the SI the following text that adds additional factors that could have plagued how we combined predictions which includes a mention that the scoring procedure could be a factor and that scoring individual questions could also be a factor:

"The factors that may have prevented our approach from producing improved predictive performance compared to past work could be because of an insufficient number of training examples. Performance could also be impacted by the method used to score predictions or that we scored individual questions rather than assign a score to a experts performance on the entire survey. Additional factors that could impact performance are the specific models that were used to combine predictions, training for experts who made predictions, and varied performance by experts over time."
