# Supplementary material for: An expert judgment model to predict early stages of the COVID-19 pandemic in the United States
Source: PLoS Comput Biol. 2022 Sep 23;18(9):e1010485. doi: 10.1371/journal.pcbi.1010485 (PMC9534428; doi:10.1371/journal.pcbi.1010485)
Supplement: Attachment — Submitted filename: ReplyToReview_expertpaper_round3__PCOMPBIOL-D-21-01928.pdf [file pcbi.1010485.s016.pdf]

Reviewer 1

I was asked to comment on the fact that "the authors have made unexplained changes to their tables which are pertinent to the critiques raised by the reviewer in the previous round (in particular SI_Redline.pdf, Table 4, Table 5)".

My comment is that the changes made seem to have been in response to the reviewer's comment that "The tables S4 and S5 are hard to interpret without some explanation of what the intercept and the beta's represent", to which the authors responded that they had removed the intercept from tables S4 and S5. However on closer inspection it looks like the values on the initial lines (which had previously been described as "Intercept" but are now described as "Expert 0") are unchanged while all other values have changed. I am guessing that the authors' code had previously erroneously used Expert 0 as the reference level that they realized the error and updated the numbers with the correct analysis but I might be wrong about this. I am happy to trust the authors' most recent analysis , although I would urge them to double-check that their latest numbers are indeed correct.

Something that may have added to the confusion is that there seem now to be two different Table S4s: one in Redline_Supporting_info.pdf ("Linear regression that compares the weights assigned to each expert using the expert-specific performance weighting and assigning experts equal weights") and the "Table S4.pdf" uploaded separately—the latter seems to be the one referred to in the manuscript on p. 3 and p. 7. So I would also urge the authors to check over all their table figure and supplementary table/figure numbers.

I don't think another round of review is required if changes of this sort are needed as this is the sort of thing that the authors can fix in proofing.

We appreciate this new reviewer for assessing our work.

The reviewer is correct. Our original table included Expert 0 as a reference level that we titled "Intercept". However, reviewer 2 raised concerns that this intercept may be difficult to interpret. In response, we removed Expert 0 as a reference in favor of a cell-means approach to Tables S4 and S5. Removing Expert 0 as a reference changed estimates for Experts1-41. We have double checked and verified that the numbers in Tables S4 and S5 are correct.

We have double checked all figure and table labels. In particular, we incorrectly labeled Table S6 (Data File) as Table S4 which we agree with the reviewer added to confusion.
